# Applications of Marine-Derived Microorganisms and Their Enzymes in Biocatalysis and Biotransformation, the Underexplored Potentials

**DOI:** 10.3389/fmicb.2019.01453

**Published:** 2019-08-20

**Authors:** Willian G. Birolli, Rafaely N. Lima, André L. M. Porto

**Affiliations:** ^1^Laboratory of Organic Chemistry and Biocatalysis, São Carlos Institute of Chemistry, University of São Paulo, São Carlos, Brazil; ^2^Center of Exact Sciences and Technology, Department of Chemistry, Federal University of São Carlos, São Carlos, Brazil

**Keywords:** marine enzymes, marine fungi, marine bacteria, biodegradation, whole cell, reduction, hydrolysis, hydroxylation

## Abstract

Biodiversity has been explored in the search for novel enzymes, including forests, savannas, tundras, deserts, and finally the sea. Marine microorganisms and their enzymes are capable of being active in high-salt concentration, large range of temperature, and high incidence of light and pressure, constituting an important source of unique biocatalysts. This review presents studies employing whole-cell processes of marine bacteria and fungi, aiming for new catalysts for different reactions in organic synthesis, such as reduction, oxidation, hydroxylation, hydrolysis, elimination, and conjugation. Genomics and protein engineering studies were also approached, and reactions employing isolated enzymes from different classes (oxidoreductases, hydrolases, lyases, and ligases) were described and summarized. Future biotechnological studies and process development should focus on molecular biology for the obtention of enzymes with interesting, fascinating and enhanced properties, starting from the exploration of microorganisms from the marine environment. This review approaches the literature about the use of marine-derived bacteria, fungi, and their enzymes for biocatalytic reactions of organic compounds, promoting a discussion about the possibilities of these microorganisms in the synthesis of different substances.

## Introduction

Microbial diversity has been explored by different researchers in the search for new biocatalysts, including the discovery of strains and enzymes with unique properties. In this quest for innovation, different environments have been approached, including forests (Pajares and Bohannan, 2016), savannas (Noriler et al., 2018), the Arctic (Malard and Pearce, 2018) and the Antarctic poles (Duarte et al., 2018), deserts (Cui et al., 2018), and finally the sea (Miao et al., 2019).

Organisms capable of growing in harsh environments can be defined as extremophiles, since their optimum metabolic activities occur under extreme conditions from a human perspective (Thompson et al., 2018). For a long time, the main interest in the marine environment, considered extreme, was the isolation and identification of natural products with biological properties, and for that, innumerous organisms and chemical structures were studied (Blunt et al., 2018).

Nowadays, researches are also focusing on the enzymes produced in the marine environment, which can present special properties (Beygmoradi et al., 2018). Assisted by sophisticated genomics, proteomics, and metabolomics analyses, the identification of novel enzymatic structures has been enhanced, including robust biocatalysts obtained by protein engineering methods (Kamble and Vavilala, 2018; Thompson et al., 2018). In addition, microbial strains isolated from several substrates, such as sediments, seawater, and mangrove detritus, have been reported as producers of enzymes with different activities, i.e., amylase, cellulase, alginate lyase, chitinase, glucosidase, inulinase, keratinase, ligninase, xylanase, and others (Bonugli-Santos et al., 2015).

Marine enzymes are capable of being active in high-salt concentration, large range of temperature, pH, organic solvents, surfactants, metal ions, and high incidence of light and pressure. Constituting a group of exceptional biocatalysts that have been applied in the biofuel, fine chemicals, pharmaceuticals, and food industries (Dalmaso et al., 2015). Additionally, enzymes obtained from cold places such as the Antarctic pole can also present activity in extreme low temperatures (Duarte et al., 2018). Therefore, these enzymes might remain active under varied operational conditions, providing competitiveness and efficiency to different industrial processes (Fernandes, 2014).

Different species of microorganisms and their enzymes are still underexplored for use in biocatalysis, including for biotransformation and biodegradation processes. However, recent achievements and the increasing number of described enzymes with interesting properties showed the potential of these biocatalysts (Nikolaivits et al., 2017). Different reactions and a large range of substrates have been employed in processes catalyzed by whole cells and isolated enzymes, expanding the application scope of marine catalysts (Birolli et al., 2015).

The marine environment represents a great opportunity for exploration of new enzymes and molecules. However, these ecosystems are already threatened by the pollution that might cause the extinction of many species of this poorly studied universe (Lima and Porto, 2016). Reducing the diversity of the sea which may present different enzymes from terrestrial organisms or similar structures with unique features for future applications (Barzkar et al., 2018).

**FIGURE 1:**
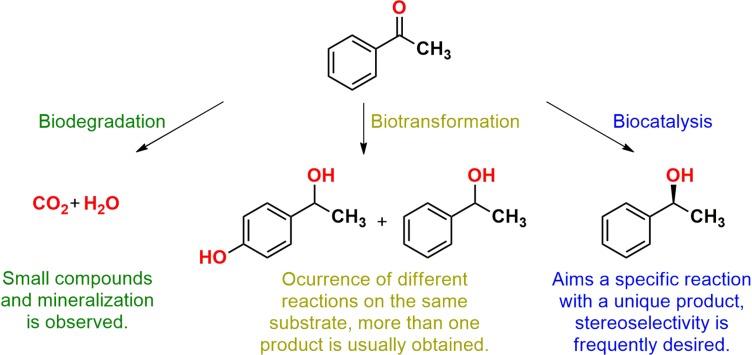
Illustration of a biodegradation, a biotransformation, and a biocatalytic reaction according to the definitions employed in this review.

Marine bacteria and fungi were used as biocatalysts in different types of processes, such as biocatalysis, in which a unique product of a specific reaction was obtained, and biotransformation, which describes several modifications on the same employed substrate, sometimes with more than one product (Parales et al., 2002). In addition, these microorganisms were also applied in biodegradation and bioremediation processes aiming for mineralization (Figure 1), but applications for decontamination already were discussed in the available literature and were not approached in this review (Nikolaivits et al., 2017).

This review aims to use marine-derived bacteria, fungi, and their enzymes for biocatalytic reactions of organic compounds, promoting a discussion about the possibilities of these microorganisms in the synthesis of different substances.

## Whole-Cell Processes

Different reactions involving whole-cell processes that employed simplified and cost-reduced procedures were presented in the literature by both bacteria and fungi catalysts (Faber, 2011).

### Bacteria

In this section, biocatalytic reactions aiming the obtention of a unique product of a specific reaction employing whole cells of bacteria were summarized. Marine-derived bacteria have been employed in different processes including kinetic resolution, deracemization, epoxidation, decarboxylation, hydroxylation, and hydrolysis reactions for the obtention of valuable compounds using new efficient biocatalysts for organic synthesis.

#### Biocatalysis

##### Kinetic resolution

**FIGURE 2:**
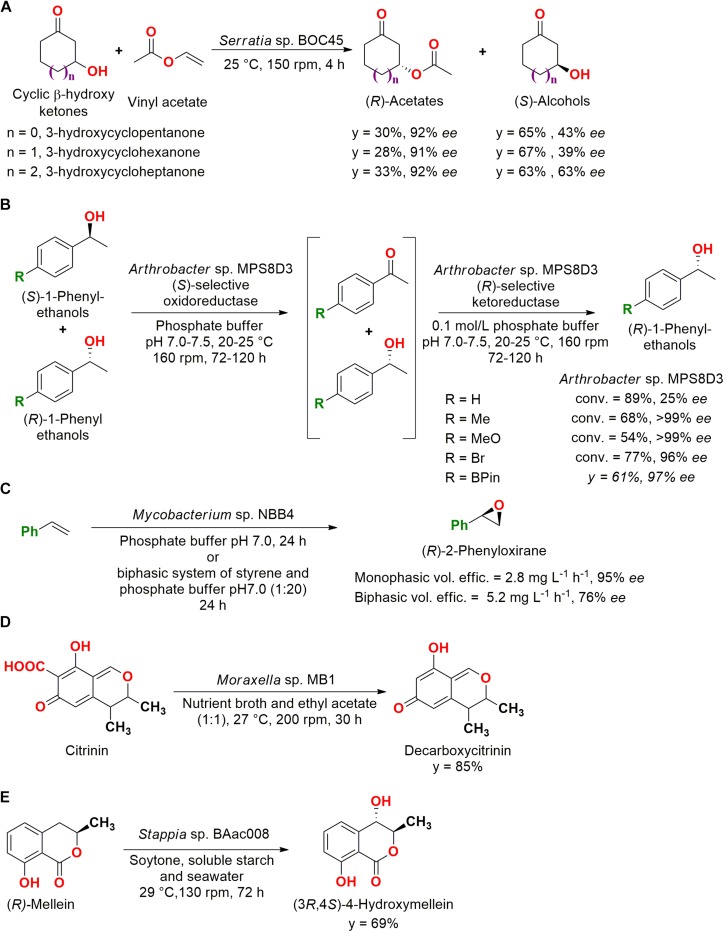
**(A)** Kinetic resolution reactions of cyclic β-hydroxy ketones by *Serratia* sp. BOC45 (Chen et al., 2017). **(B)** Deracemization of 1-phenylethanols, including heteroatoms, by *Arthrobacter* sp. MPS8D3 (Palmeira et al., 2014). **(C)** Enantioselective epoxidation of styrene by *Mycobacterium* sp. NBB4 (Cheung et al., 2013). **(D)** Decarboxylation of citrinin to decarboxycitrinin by the marine bacteria *Moraxella* sp. MB1 (Devi et al., 2006). **(E)** Hydroxylation of (*R*)-mellein to (3*R*,4*S*)-4-hydroxymellein by *Stappia* sp. BAac008 (Feng et al., 2010).

For example, cyclic β-hydroxy ketones were employed in a kinetic resolution by whole cells of 26 strains isolated from marine sediments. Seven bacterial strains presented enantioselectivity for (*R*)- and (*S*)-3-hydroxycyclopentanone, (*R*)- and (*S*)-3-hydroxycyclohexanone, and (*R*)- and (*S*)-3-hydroxycycloheptanone (Figure 2A) (Chen et al., 2017).

The kinetic resolution is the most common process for the obtention of separated enantiomers. However, this reaction is limited to a yield of 50%, since only one of the enantiomers is transformed. Meanwhile, deracemization can achieve 100% yield involving a selective oxidation into a prochiral intermediate, which is subsequently reduced to the desired single enantiomer (Nasario et al., 2016).

##### Deracemization and reduction

In an application of a deracemization system, microorganisms isolated from sediments of the Admiralty Bay (King George Island, Antarctic) were screened under low temperatures. From 232 psychrophile/psychrotroph microorganisms originally isolated, 15 bacterial strains contained oxidoreductases capable of performing deracemization in 1-(4-methyl-phenyl)ethanol (conv. = 54–89%, 25–99% *ee S*-enantiomer). It is important to note that the microorganisms were unable to carry out the reaction over 30°C (Araujo et al., 2011). In a subsequent study, derivatives of 1-phenylethanol containing heteroatoms like tin, silicon, phosphorous, and boron were also employed in the catalysis by *Arthrobacter* sp. MPS8D3 (*y* = 59–71%, 42–97% *ee S*-enantiomer), in which the presence of oxygen favored the deracemization (Palmeira et al., 2014). The reactions are presented in Figure 2B.

In another work, 33 bacterial strains isolated from the Eastern Mediterranean Sea in a deep hypersaline anoxic basin (DHAB) were employed for the stereoselective reduction of propyl *anti*-2-oxotricyclo[2.2.1.0]heptan-7-carboxylate, an important intermediate for the synthesis of prostaglandin (phosphate buffer pH 7.2, 30°C, 7 h). *Halomonas aquamarina* 9B enantioselectively hydrolyzed the substrate producing *S*-*anti*-2-oxotricyclo[2.2.1.0]heptan-7-carboxylic acid with *y* = 55% and 81% *ee*, whereas the unreacted substrate presented 99% *ee*. On the other hand, *Bacillus horneckiae* 15A carried out a high stereoselective reduction obtaining the propyl *S*-*anti*-2-hydroxytricyclo[2.2.1.0]heptan-7-carboxylate with *y* = 56% and 87% *ee*, while the remaining substrate presented 99% *ee* (De Vitis et al., 2015).

##### Epoxidation

Marine bacteria were also employed for the production of enantioenriched epoxides, such as the *Mycobacterium* NBB4 isolated from estuarine sediments at Garrison Point reserve (Sydney, NSW, Australia) and deposited as JCM 17769. When grown in the presence of ethene, this strain was able to epoxidize a wide range of alkenes, including terminal, cyclic, aromatic, and functionalized substrates (propene, 1-butene, 1-hexene, 1-octene, 1-decene, cyclopentene, cyclohexene, styrene, indene, allyl alcohol, dihydropyran, and isoprene) with conv. = 25–81%. Moreover, styrene was employed in an enantioselectivity assessment in a monophasic and biphasic system with interesting results (Figure 2C), showing that marine bacteria can be employed for epoxidation reactions (Cheung et al., 2013).

##### Hydrolysis

Hydrolysis reactions, in which water molecules are used for breaking molecular bonds, have been carried out employing marine bacteria, including the hydrolysis of polysaccharides and nitriles (Brandao and Bull, 2003; Rodrigues et al., 2017).

A high-throughput screening in solid medium was employed in the search for inulinase, amylase, esterase, and lipase activities in new marine bacteria (205 isolates) from Azores, Portugal (strains isolated from intertidal shallow-water hydrothermal vents, intertidal pools, and open waters). The highest inulinase activity (catalysis of the endohydrolysis of 2,1-β-D-fructosidic linkages in inulin) was presented by *Bacillus subtilis* 83, showing that the bacterial strains from this unique environment might be successful biocatalysts for biotechnological processes (Rodrigues et al., 2017).

For the hydrolysis of nitriles, actinomycetes isolated from deep-sea sediments were employed for reaction with aliphatic and aromatic compounds (acetonitrile, propionitrile, acrylonitrile, butyronitrile, succinonitrile, valeronitrile, isovaleronitrile, benzonitrile, acetamide, and benzamide) with the substrate as sole carbon source (phosphate buffer pH 7.0, 30°C, 200 rpm). From the seven strains of marine bacteria (*Rhodococcus erythropolis* 870-AN019, *Rhodococcus equi* 871-AN029, *R. equi* 871-AN030, *Rhodococcus opacus* 871-AN040, *R. erythropolis* 871-AN053, *R. erythropolis* 67-BEN001, and *R. erythropolis* 122-AN065), six presented constitutive nitrile hydratase and amidase enzymes, showing higher activity and broader specificity than the terrestrial strains employed in the same study. It is noteworthy that the hydrolysis rate was determined by the ammonia release (Brandao and Bull, 2003).

##### Decarboxylation

In another type of reaction, a decarboxylation, *Moraxella* spp. MB1 isolated from the seaweed *Bryopsis plumosa* collected from Malvan (Goa coast, India) was screened from six strains and employed in the production of decarboxycitrinin from the toxin citrinin, maintaining the antibiotic activity of the substrate (Figure 2D). The reaction was performed by an intracellular decarboxylase in a biphasic system composed of nutrient broth and ethyl acetate (1:1), since the biocatalyst presented the useful feature of producing a unique product and tolerated the presence of organic solvents (Devi et al., 2006).

##### Hydroxylation

A unique product was also obtained in the employment of *Stappia* sp. BAac008, isolated from the green alga *Enteromorpha compressa*, in the hydroxylation reaction of (*R*)-mellein (Figure 2E). The substrate was a natural product isolated from a marine fungus *Cladosporium* sp. and employed for the production of (3*R*,4*S*)-4-hydroxymellein, which presented mild antibacterial activity against antibiotic-resistant *Staphylococcus aureus*. The *ee* of the product was not presented (Feng et al., 2010).

The natural product Terreusinone, an ultraviolet-A (UV-A)-protecting dipyrroloquinone isolated from the marine fungus *Aspergillus terreus*, was employed in a hydroxylation reaction by the marine actinomycete *Streptomyces* sp. MFAac18, which was obtained from the sea plant *Zostera marina* obtained at Bijin Island (Gyeongnam, Korea). This biotransformation of terreusinone resulted in terreusinol (soytone, soluble starch, and seawater, 29°C, 35 days, static culture, *y* = 24%), which presented high values of UV-A protection, enabling different applications in the cosmetic industry (Li et al., 2003).

Another strain of the same genus, *Streptomyces* sp. MFAac67, also isolated from the sea plant *Z. marina* (Jeju Island, Korea) was employed in a hydroxylation reaction of the sesquiterpene cyclonerodiol, generating the stereoisomers (*Z*)- and (*E*)-cyclonerotriol with yields of 16 and 35%, respectively (soytone, soluble starch, and seawater, 29°C, 130 rpm, 14 days) (Li et al., 2007). These results indicated that marine strains of the *Streptomyces* genus can be important biocatalysts for hydroxylation reactions.

These studies emphasized the importance of carrying out a screening of strains for a successful identification of new biocatalysts, since several strains were not able to perform the desired reactions, as observed for the kinetic resolution of β-hydroxy ketones (Chen et al., 2017). Moreover, the use of different bacterial strains promoted different reactions, as determined for the substrate propyl *anti*-2-oxotricyclo[2.2.1.0]heptan-7-carboxylate (De Vitis et al., 2015), and enzymatic activities, as inulinase (Rodrigues et al., 2017).

In addition, the reported results showed interesting and unique properties presented by marine-derived bacteria, justifying and encouraging the search and screening of these microorganisms for several reactions with a large range of substrates.

#### Biotransformation

A considerable number of biotransformation reactions, in which different modifications were performed in the same substrate, were carried out employing marine bacteria, differentiating these studies from reactions of biocatalysis that report a specific transformation with a well-defined product (Parales et al., 2002).

For example, cholesterol (initial concentration of 0.5 mg ml^–1^) was biotransformed to cholest-4-ene-3,6-dione by an organic-tolerant *Bacillus* sp. BC1 isolated from sediment of the Arabian Sea. The reaction was performed in 50% (v/v) chloroform in mineral salt medium (pH 7.0, 28°C, orbital stirring, 24 h), showing the possibility of a faster steroid transformation by marine microorganisms in the presence of organic solvents than in aqueous media, probably because of the lipophilicity of the substrate (Sardessai and Bhosle, 2003).

**FIGURE 3:**
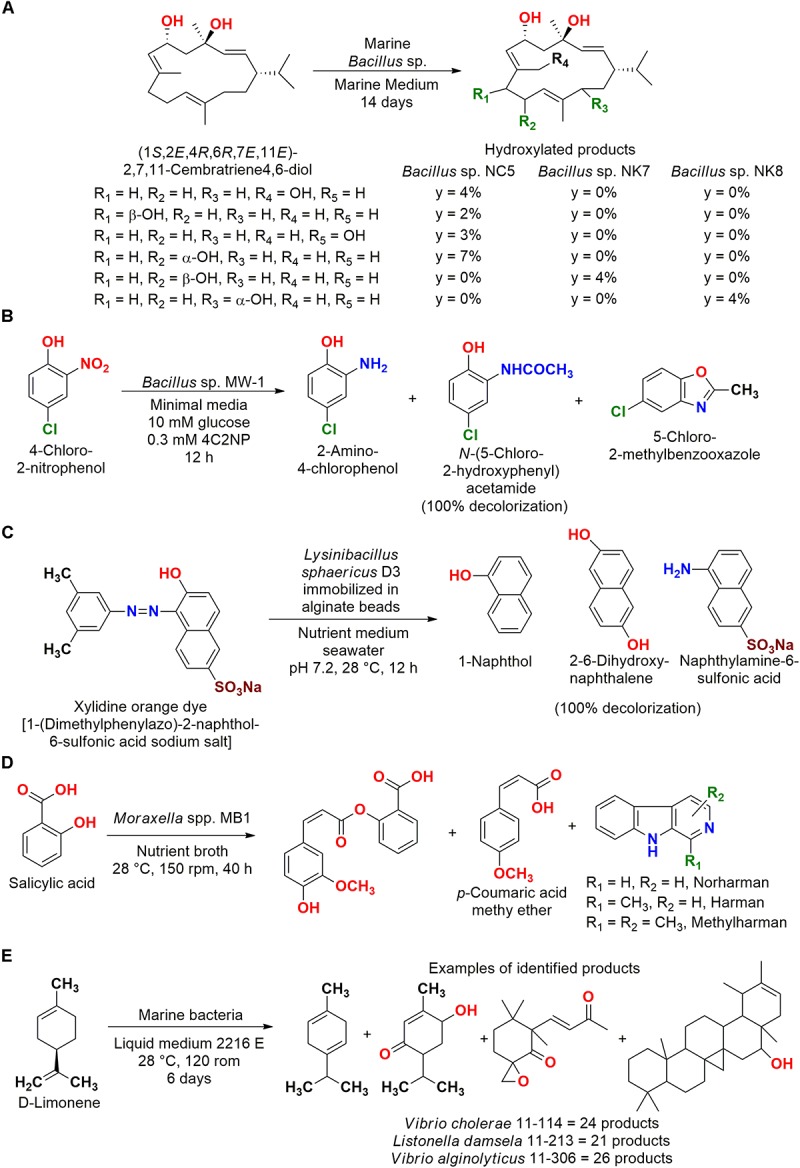
**(A)** Biotransformation of (1*S*,2*E*,4*R*,6*R*,7*E*,11*E*)-2,7,11-cembratriene-4,6-diol to hydroxylated products by *Bacillus* sp. strains (El Sayed et al., 2008). **(B)** Biotransformation of 4-chloro-2-nitrophenol to different products in sequential steps by *Bacillus* sp. MW-1 (Arora and Jain, 2012). **(C)** Decolorization of xylidine orange dye by *L. sphaericus* D3 (Devi et al., 2017). **(D)** Biotransformation of salicylic acid by *Moraxella* spp. MB1 (Wahidullah et al., 2013). **(E)** Biotransformation of D-limonene by *Vibrio cholerae* 11–114, *Listonella damsela* 11–213, and *Vibrio alginolyticus* 11–306 (Li et al., 2006).

In another biotransformation reaction, the marine bacteria *Bacillus* sp. NK7, *Bacillus* sp. NK8, and *Bacillus* sp. NC5 isolated from the Red Sea sponge *Negombata magnifica* were able to biotransform (1*S*,2*E*,4*R*,6*R*,7*E*,11*E*)-2,7,11-cembatriene-4,6-diol (Figure 3A). Hydroxylated compounds that retained the anti-invasive activity against the human metastatic prostate cancer without the toxicity of the substrate were obtained, showing the importance of these biocatalysts for the production and discovery of new organic compounds with interesting biological activities (El Sayed et al., 2008).

Some reactions were carried out with larger substrates, such as in the biotransformation of naphthalene and methyl naphthalene to (2-naphthylmethyl)succinic acid by the anaerobic sulfate-reducing *Desulfobacteraceae* NaphS3 and NaphS6 (anoxic HCO_3_^–^/CO_2_^–^ buffered sulfide-reduced artificial seawater medium, 8-heptamethylnonane, 28°C, 100 rpm, 10–80 days). These strains belong to the class *Deltaproteobacteria* and were isolated from Mediterranean marine sediment employing naphthalene as the sole carbon source. In this study, a large assumed subunit of (2-naphthylmethyl)succinate synthase was proposed based on gene sequence similarity. It is noteworthy that conversions were not reported (Musat et al., 2009).

Biotransformations by reduction reactions were also performed, including by obligate marine bacteria such as *Shewanella marisflavi* EP1, which was employed in the biotransformation of 2,4-dinitrotoluene with 100% of conversion (basal medium, lactate as electron donor, pH 7.0–9.0, 4–40°C, 2–8% NaCl, 24 h). The obtained products were 2-amino-4-nitrotoluene, 4-amino-2-nitrotoluene, and 2,4-diaminotoluene. In a process that employed lactate as electron donor and the substrate as electron acceptor in a reduction reaction involving cytochromes, dehydrogenases, menaquinone, and flavins (Huang et al., 2015).

In a biotransformation process, when different reactions occur in the substrate producing small molecules and mineralization to water and CO_2_, a biodegradation process takes place. The frontiers separating these two phenomena are not very clear, but the difference between them lies in the focus of the study and the degree of substrate modification (Parales et al., 2002).

In the biotransformation of carbazole, for example, the small-molecule anthranilic acid was obtained in the reaction by marine bacterial strains isolated from seawater of different Japanese areas. These strains were screened (0.3 mg L^–1^ of initial concentration, natural seawater medium, 30°C, 3 days) by decolorization of the substrate, and 11 isolates closely related to the *Caulobacter*, *Erythrobacter*, *Sphingosinicella*, *Hyphomonas*, *Kordiimonas*, and *Lysobacter* genera were characterized. These microorganisms biotransformed carbazole to anthranilic acid, a harmless and easily biodegradable compound. However, conversions were not reported (Maeda et al., 2009).

A sequence of reactions that characterize a biotransformation/biodegradation process was observed for 4-chloro-2-nitrophenol (Figure 3B). A total of 82 strains isolated from sediment, water, and sponge particles at 30 m depth in the Bay of Bengal (India) were screened by decolorization. *Bacillus* sp. MW-1 consumed 100% of the substrate in co-metabolism with glucose, since this strain did not biodegrade this compound as sole carbon source (Arora and Jain, 2012).

An example of biodegradation study involving marine bacteria was the use of *Sphingomonas* sp. 2MPII, which was isolated from polluted sediment near a refinery in the Gulf of Fos (France), in the degradation (seawater, salts, and vitamins, 20–25°C, 70 rpm, 8–12 h) of polycyclic aromatic hydrocarbons (PAHs). Including phenanthrene, 2-methylphenanthrene (2-MP), 9-methylphenanthrene (9-MP), and dibenzothiophene. These compounds were employed as sole carbon source and experiments with more than one substrate at the same reaction were carried out. The biodegradation rates increased or decreased, depending on the PAHs combination. For example, 64% of 2-MP was biodegraded employing only this substrate, but in experiments with both 9-MP and 2-MP, an 83% biodegradation of 2-MP was determined (Nadalig et al., 2002).

The substrate consumption in biotransformation/biodegradation reactions can be determined by different methods. In the employment of *Sphingomonas* sp. 2MPII for PAHs biodegradation, the substrate biotransformation was measured by substrate disappearance and by oxygen uptake in sole carbon experiments, characterizing the reported results as a biodegradation study that indirectly determined the degree of mineralization (Nadalig et al., 2002). Whereas the consumption of xylidine orange dye was determined by decolorization and confirmed by the presence of the identified metabolites (Devi et al., 2017).

Biotransformation/biodegradation products were also presented in the employment of *Lysinibacillus sphaericus* D3 isolated from the sponge *Gelliode scellaria*, which was obtained at Mandapam in the Indian coast. This biocatalyst was immobilized in sodium alginate beads for decolorization of the xylidine orange dye 1-(dimethylphenylazo)-2-naphthol-6-sulfonic acid sodium salt (Figure 3C). A decolorization of 100% was observed, and the metabolites naphthol, 1-naphthylamine-6-sulfonic acid, 2-6-dihydroxynaphthalene, and a dye dimer were identified (Devi et al., 2017).

Different studies reported biotransformation/biodegradation processes and described the obtained products. Another example was the use of the marine bacteria *Alteromonas macleodii* BP-PH, *Neptunomonas naphthovorans* CAR-SF, and *Cycloclasticus pugetii* DBF-MAC isolated from seawater (Japan) for biodegradation of carbazole, dibenzofuran, and biphenyl. Metabolites were detected and identified: 2-hydroxy-6-oxo-(2-aminophenyl)hexa-2,4-dienoic acid for carbazole; 4-(3-methoxy-2-benzofuranyl)-2-oxo-3-butenoic acid, (chroman-4-on-2-yl)acetic acid, and salicylic acid for dibenzofuran; and benzoic acid for biphenyl. The conversions were not presented (Fuse et al., 2003).

Marine bacteria from the *Alcaligenes* genus (*Alcaligenes faecalis* KT336452, KT336455, KT354279, and KT336456) were also employed in this type of process and biotransformed the recalcitrant and carcinogenic dye Congo Red with complete decolorization (100% conversion, Luria–Bertani broth, 120 rpm, 37°C, 48 h). Different compounds and intermediates were identified by liquid chromatography–mass spectrometry (LC-MS) and a partial biodegradation pathway was proposed, including the biotransformation products 4,4′-diaminobiphenyl, 1,2′-diaminonapthalene-4-sulfonic acid, 4-nitroso-1,1′-biphenyl, sodium naphthalen-1-olate, and 3,4-dinitroso-naphthalene-1-sulfonic acid, showing the efficiency of this process (D’Souza et al., 2017).

In some studies aiming biotransformation, the production of more complex compounds employing the substrates as construction blocks in biosynthesis reactions were observed. For example, *Moraxella* spp. MB1 isolated from the seaweed *Bryopsis plumosa* collected from Malvan (Goa coast, India) was employed for the biodegradation of salicylic acid, a metabolite of analgesics (Figure 3D). In this study, more complex metabolites than the employed substrate were identified by electrospray ionization (ESI)-MS/MS. Moreover, the biotransformation of salicylic acid to cinnamates occurred without ring cleavage to catechol, which is the metabolic pathway usually observed in the biodegradation of this compound (Wahidullah et al., 2013).

In another study, bacterial strains isolated from sediment of the Daya Bay (China) were employed in the biotransformation of D-limonene, and several more elaborated compounds were identified (Figure 3E). Showing that not only products of biotransformation were detected, but also microbial metabolites related to the biosynthetic pathways of terpenoids, which might have been induced. It is important to note that a control reaction without limonene was carried out and these compounds were not observed (Li et al., 2006).

Computerized approaches for the use of marine biocatalysts were also performed, i.e., a bioinformatic study identified 265 strains of *Actinobacteria* and *Proteobacteria* as steroid degraders, including marine strains. Some of them were subsequently confirmed in biodegradation experiments. This study emphasized the contribution and importance of marine strains for understanding ecological and evolutionary aspects of microbiology (Bergstrand et al., 2016).

In biotransformation studies, it is important to note that the involved reactions should be evaluated, including the characterization of transient and possible intermediates. Moreover, conversion and yield must be determined for a better understanding of the process and comparison of the described study with the available information of the literature. It is also important to note that secondary products should be identified and quantified.

In summary, marine-derived bacteria were employed in whole-cell processes of biocatalysis, biotransformation, and biodegradation, showing the versatility of these biocatalysts. However, it was observed that these marine microbes were poorly studied considering the environmental diversity and importance of the sea.

### Fungi

Fungi from marine environment were employed in different biocatalytic processes aiming the obtention of specific products. In addition, the desired compounds should present the required stereochemistry in some studies. These microorganisms were employed as biocatalysts for different reactions, including reduction, oxidation, hydroxylation, hydrolysis, kinetic resolution, and conjugation.

#### Reduction

Marine-derived fungi were employed as biocatalyst in reduction reactions of carbonyl groups, C–C double bonds, and carboxylic acids.

##### Reduction of carbonyl groups

Different fungal strains were employed in reduction reactions for obtention of enantioenriched compounds, such as in the screening of nine strains isolated from marine sponges and Cnidarian species at São Sebastião (São Paulo, Brazil) for the enantioselective reduction of 1-(4-methoxyphenyl)ethanone to 1-(4-methoxyphenyl)ethanol (*y* = 15–95%, 69–99% *ee* for *S* or *R*-enantiomer, phosphate buffer solution pH 7.0, 32°C, 120 rpm, 3–9 days). Prelog or anti-Prelog products were obtained depending on the employed fungi, including reactions with > 99% *ee* of the *S*-enantiomer and > 99% *ee* of the *R*-enantiomer for *Aspergillus sclerotiorum* CBMAI 849 and *Bionectria* sp. Ce5, respectively, showing that these strains might be an important source of new reductases (Rocha et al., 2012b).

**FIGURE 4:**
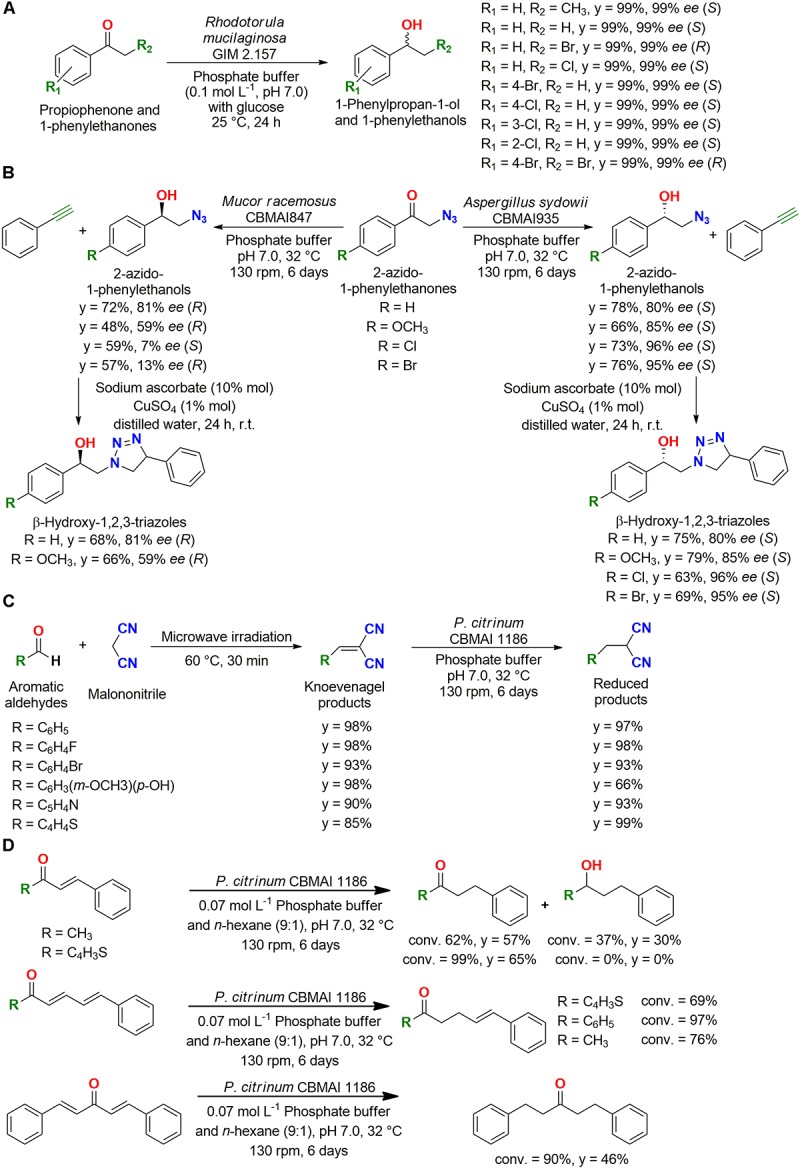
**(A)** Reduction of 1-phenylpropan-1-one and 1-phenylethanones to 1-phenylpropan-1-ol and 1-phenylethanols by *Rhodotorula mucilaginosa* GIM 2.157 (Liu et al., 2018b). **(B)** Enantiocomplementary synthesis of β-hydroxy-1,2,3-triazoles employing marine-derived fungi as biocatalysts (Alvarenga and Porto, 2017). **(C)** Reduction of double bonds of Knoevenagel condensation adducts by *P. citrinum* CBMAI 1186 (Jimenez et al., 2016). **(D)** Reduction of C**–**C double bonds of α,β-, di-α,β-, and mono-α,β,γ,δ-unsaturated ketones by *P. citrinum* CBMAI 1186 (Ferreira et al., 2015b).

*Rhodotorula mucilaginosa* GIM 2.157 was successfully employed for the reduction of nine derivatives of 1-phenylpropan-1-one and 1-phenylethanones to 1-phenylpropan-1-ol and 1-phenylethanols, respectively, with 99% yield and 99% *ee* for the *S*- or *R*-enantiomer depending on the substrate (Figure 4A). In addition, the reusability of the cells was evaluated for reduction of propiophenone; *R. mucilaginosa* GIM 2.157 resulted in over 95% yield with 99% *ee* for three cycles, and *Rhodotorula rubra* AS 2.2241 gave the same results (95% yield with 99% *ee*) for nine cycles (Liu et al., 2018b).

A faster procedure that deserves emphasis was reported by the employment of growing cells in the reduction of nine derivatives of 1-phenylethanones with different groups in the aromatic ring (R = 2-Br, R = 3-Br, R = 4-Br, R = 2-NO_2_, R = 3-NO_2_) also by *R. rubra* AS 2.2241, simplifying the steps for preparation of the biocatalyst and reducing the costs of a future industrial scale. In addition, yields of 99% with 99% of *ee* for the *S*-enantiomer were obtained for these compounds in the employed experimental conditions (RM1 medium pH 7.0, growing cells, 28°C, 220 rpm, 72 h), whereas five tested substrates (R = 4-OCH_3_, R = 4-CH_3_, R = 4-CH_2_CH_3_, R = 2-Cl and 4-Cl, and R = 3-Cl and 4-Cl) did not react (Liu et al., 2018a).

In a recent study, α-chloroketones obtained from 1-phenylethanones employing oxone^®^ and NH_4_Cl (*y* = 90–97%, methanol, 70°C, 60 min) were reduced by different marine-derived fungi to produce enantioenriched chlorohydrins (conv. = 12–97%, phosphate buffer pH 7.0, 32°C, 130 rpm, 6 days). It is important to note that the biocatalytic reactions were carried out after the filtration of the ozone salt and methanol removal by rotoevaporation, thus without purification of the intermediate in a greener methodology (Morais et al., 2018). It is noteworthy that these studies reported different advances in the use of marine-derived fungi as biocatalysts, generating cleaner, easier, and cost-reduced processes.

Marine-derived strains of fungi (*A. sclerotiorum* CBMAI 849, *Cladosporium cladosporioides* CBMAI 857, *Penicillium raistrickii* CBMAI 931, and *Penicillium citrinum* CBMAI 1186) also isolated from marine sponges at São Sebastião (São Paulo, Brazil) were also employed in the reduction of different aromatic ketones, such as α-keto azides to β-azidophenylethanols with different groups in the aromatic ring (R = 4-H, 4-OCH_3_, 4-Br or 4-NO_2_). The reactions were carried out with excellent yields and enantioselectivities (*y* = 0–93%, 28–99% for the *R*- or *S*-enantiomer, phosphate buffer solution pH 7.0, 32°C, 130 rpm, 9 days), which were determined as Prelog and anti-Prelog products depending of the substrate and the employed biocatalyst (Rocha et al., 2015).

Then, the enantiocomplementary biocatalysts *Aspergillus sydowii* CBMAI 935 and *Mucor racemosus* CBMAI 847 were used in the reduction of 2-azido-1-phenylethanols, which were then employed in the synthesis of β-hydroxy-1,2,3-triazoles with CuSO_4_ and sodium ascorbate in aqueous medium (Figure 4B). This study showed an interesting approach employing biocatalysis and click chemistry to obtain these molecules by a greener method (Alvarenga and Porto, 2017).

Another example of carbonyl reduction of compounds containing nitrogen atoms was the production of dioxindole from isatin, in reactions carried out by marine-derived fungi isolated from the Atlantic Ocean, Brazil (*Aspergillus* sp. CBMAI 1829, *Acremonium* sp. CBMAI 1676, *Westerdykella* sp. CBMAI 1679, and *A. sydowii* CBMAI 935). These experiments showed that the reduction of nitrogenated compounds, which are usually consumed as nutrient source in biocatalytic processes by whole cells, could be performed (*y* = 26–37%, 18–66% *ee S*-enantiomer, phosphate buffer pH 7.0, 32°C, 130 rpm, 3–7 days) (Birolli et al., 2017).

The strain *P. citrinum* CBMAI 1186 employed for the reduction of 1-phenylethanones (Rocha et al., 2012b) was also used as immobilized biocatalyst on silica gel, silica xerogel, and chitosan for the production of 1-phenylethanol (*y*_free cells_ = 35%, 69% *ee S*-enantiomer, *y*_chitosan_ = 95%, 99% *ee S*-enantiomer, *y*_silica gel_ = 0%, *y*_silica xerogel_ = 0%) and 2-chloro-1-phenylethanol (*y*_free cells_ = 66%, 31% *ee S*-enantiomer, *y*_chitosan_ = 98%, 0% *ee S*-enantiomer, *y*_silica gel_ = 62%, 25% *ee S*, *y*_silica xerogel_ = 0%). It was important to note that the employed support strongly influenced the reaction, promoting different results under the same experimental conditions (phosphate buffer pH 7.0, 9 days, 120 rpm, 32°C) (Rocha et al., 2012a).

##### Reduction of C–C double bonds

Fungi, including terrestrial (Romagnolo et al., 2016a) and marine-derived strains (Birolli et al., 2015), were also successfully employed in the reduction of C–C double bonds. For example, microwave irradiation and whole-cell catalysis were employed in the synthesis of aromatic malononitrile derivatives. A Knoevenagel condensation reaction under microwave irradiation was carried out, followed by a C–C double bond reduction in excellent yields by the same marine-derived fungus *P. citrinum* CBMAI 1186 employed for carbonyl reduction (Figure 4C), showing that these two green protocols can be employed together in organic synthesis (Jimenez et al., 2016).

The promising results of free cells of *P. citrinum* CBMAI 1186 as biocatalyst for reduction reactions stimulated further studies of immobilization. This strain was employed both free (*y* = 20%) and immobilized on low-cost biopolymers (cotton, fibroin, and kapok, *y* = 68–75%) in the chemoselective reduction of chalcones to dihydrochalcones in good yields. The immobilization enabled an easy separation of the catalyst from the reaction media and increased its activity even after storage at 4°C (*y*_free cells_ = 8%, *y*_immobilized =_ 19–27%), showing that this biocatalyst can also be employed immobilized on natural fibers (Ferreira et al., 2014).

In subsequent studies, *P. citrinum* CBMAI 1186 was employed in a biphasic system for reduction of double bonds of α,β-, di-α,β-, and mono-α,β,γ,δ-unsaturated ketones, which are larger substrates (Figure 4D). The observed chemoselective reduction of the α,β-C–C double bond in α,β-unsaturated ketones was an interesting result, since it would be very difficult to obtain this transformation using conventional methods. For bis-α,β-unsaturated ketones, four different compounds resulted from different double bonds (major product) and carbonyl reductions, presenting the substrate broad range of this marine biocatalyst (Ferreira et al., 2015a, b).

##### Reduction of carboxylic acids

Filamentous fungi from terrestrial and marine environments were screened for the reduction of methyl cinnamate to 2-phenoxyethanol. In this selection, the strains *Gliomastix masseei* MUT 4855, *Penicillium vinaceum* MUT 4892, *Coprinellus* sp. MUT 4897, and *P. citrinum* MUT 4862 isolated from marine algae did not catalyze the desired reaction, whereas *Syncephalastrum racemosum* MUT 2770 isolated from air completely reduced the substrate to 2-phenoxyethanol in 7 days (25°C, 110 rpm, malt extract medium) (Romagnolo et al., 2016a).

As described in the literature, *P. citrinun* CBMAI 1186 was promising in the reduction of C–C double bonds, but this species represented by the strain *P. citrinun* MUT 4862 was not adequate for this reduction of a carboxylic acid (Jimenez et al., 2016; Romagnolo et al., 2016b). These results emphasized the importance of a screening for reactions employing whole cells. However, it is important to note that different species were employed and not different strains of the same species from marine and terrestrial environment. Therefore, the carboxylic acid reductase can be characteristic of the employed fungus species.

#### Hydroxylation

**FIGURE 5:**
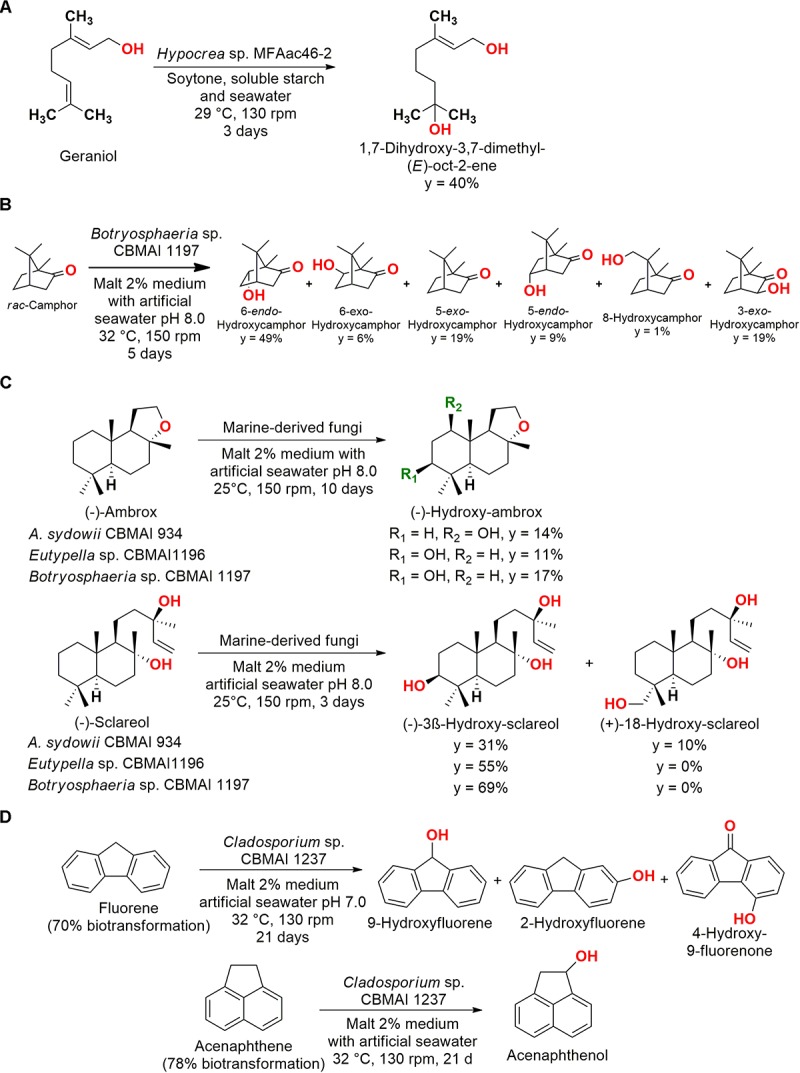
**(A)** Hydroxylation of geraniol by *Hypocrea* sp. MFAac46-2 (Leutou et al., 2009). **(B)** Hydroxylation of *rac*-camphor by *Botryosphaeria* sp. CBMAI 1197 (De Jesus et al., 2017). **(C)** Biotransformation of (–)-Ambrox and (–)-Sclareol by marine-derived fungi (Martins et al., 2015). **(D)** Biotransformation of fluorene and acenaphthene by *Cladosporium* sp. CBMAI1237 (Birolli et al., 2018).

Marine-derived fungi were employed in hydroxylation reactions of different substrates. For example, geraniol (a precursor of monoterpenes) was hydroxylated to 7-dihydroxy-3,7-dimethyl-(*E*)-oct-2-ene by the marine fungus *Hypocrea* sp. MFAac46-2 (Figure 5A), which was isolated from the brown marine and edible alga *Undaria pinnatifida* at South Korea (Leutou et al., 2009).

In another example, the fungus *Botryosphaeria* sp. CBMAI 1197 isolated from the marine alga *Bostrychia radicans* (São Sebastião, São Paulo, Brazil) was employed in the hydroxylation of racemic camphor (Figure 5B). The major product was 6-*endo*-hydroxycamphor, but the biotransformation process resulted in different monohydroxylated compounds. It is important to note that these modifications in sp^3^ carbons are difficult to be performed by conventional methods (De Jesus et al., 2017).

More complex compounds were also subjected to hydroxylation by marine fungi. For (−)-ambrox, (−)-sclareol, and (+)-sclareolide, nine marine-derived strains of fungi were screened. Four strains, *Eutypella* sp. CBMAI 1196, *A. sydowii* CBMAI 934, *Botryosphaeria* sp. CBMAI 1197, and *Xylaria* sp. CBMAI 1195, were able to carry out regioselective reactions, obtaining mono-hydroxylated products (Figure 5C). For the obtention of (−)-3β-hydroxy-sclareolide from (+)-sclareolide employing *Eutypella* sp. CBMAI1196 and *Botryosphaeria* sp. CBMAI 1197, yields of 7 and 34% were observed, respectively (malt 2% medium with artificial seawater, pH 8.0, 25°C, 150 rpm, 12 days) (Martins et al., 2015).

In a screening of marine fungi with PAHs, such as fluorene and acenaphthene, different hydroxylated products of biotransformation were observed (Figure 5D). In addition, anthracene (71% biotransformation), fluoranthene (52% biotransformation), pyrene (62% biotransformation), and nitropyrene (64% biotrans- formation) were biotransformed/biodegraded into anthraquinone in a multistep reaction (malt 2% medium with artificial seawater, pH 7.0, 32°C, 130 rpm, 21 days). The most efficient strain *Cladosporium* sp. CBMAI1237 was able to transform anthracene in the presence and absence of artificial seawater (42 and 26%, respectively, for 14 days of reaction), but a increased biotransformation was observed in high salts concentration, showing the importance of the evaluation of minerals addition for marine-derived strains (Birolli et al., 2018).

#### Oxidation, Hydrolysis, and Kinetic Resolution

**FIGURE 6:**
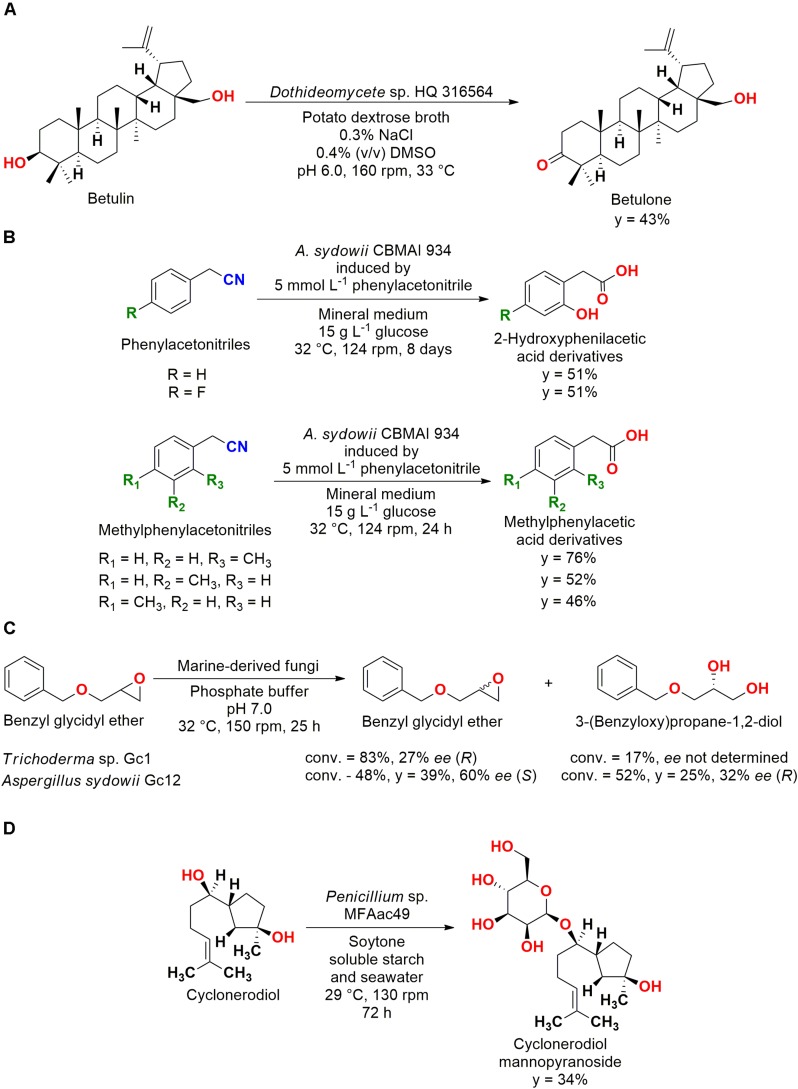
**(A)** Oxidation of betulin to betulone by *Dothideomycete* sp. HQ 316564 (Liu et al., 2013). **(B)** Hydrolysis of phenylacetonitrile derivatives to 2-hydroxyphenylacetic acids by *A. sydowii* CBMAI 934 (De Oliveira et al., 2013, 2014). **(C)** Kinetic resolution of benzyl glycidyl ether by *A. sydowii* Gc12 and *Trichoderma* sp. Gc1 (Martins et al., 2011). **(D)** Conjugation and biotransformation of cyclonerodiol by *Penicillium* sp. MFAac49 (Li et al., 2007).

Oxidation reactions were also performed by marine-derived fungi. The *Dothideomycete* sp. HQ 316564 was employed in the obtention of betulone from betulin in a regiospecific reaction. The reactional conditions were optimized, and this new biocatalytic approach was established employing mild conditions (Figure 6A), therefore expanding the number of applications of marine fungi for oxidation reactions (Liu et al., 2013).

Another interesting application of these microorganisms was the hydrolysis of phenylacetonitriles by *A. sydowii* CBMAI 934 for the obtention of 2-hydroxyphenylacetic acid. Subsequently, this strain was also employed for the hydrolyses of 4-fluorophenylacetonitrile and methylphenylacetonitriles (Figure 6B). These studies showed that *A. sydowii* CBMAI 934 can be an important green catalyst and source of enzymes for nitrile hydrolyses (De Oliveira et al., 2013, 2014).

Finally, a kinetic resolution reaction was also carried out. The complementary enantioselectivity (±)-for the opening of the epoxide ring of the substrate 2-(benzyloxymethyl)oxirane (benzyl glycidyl ether) by *A. sydowii* Gc12 (later deposited as CBMAI 933) and *Trichoderma* sp. Gc1 (later deposited as CBMAI 932) was reported (Figure 6C). These fungal strains were isolated from marine sponges of the South Atlantic Ocean (Brazil), showing biocatalytic potential for a wide variety of reactions (Martins et al., 2011).

#### Conjugation

Biotransformation/biodegradation processes by fungal strains from marine environment were reported in different studies. A fascinating example of conjugation reactions that are frequently observed in biodegradation processes (Alvarenga et al., 2014) was reported for the bioactive sesquiterpene, cyclonerodiol. The fungal strain *Penicillium* sp. MFAac49 isolated from the brown alga *Sargassum thunbergii* collected at Songjeung Beach of Busan conjugated the substrate to the glycosidic metabolite 7-*O*-(β-D-mannopyranosyl)cyclonerodiol (Figure 6D) (Li et al., 2007).

The literature about biodegradation processes employing marine microorganisms were not the focus of this review. However, an interesting study that shows the potential of marine-derived fungi for bioremediation processes approached the biodegradation of oil spills. Fungi strains isolated from a contaminated site at the Mediterranean Sea (67 strains from water and 17 from sediments) were assessed by the use of crude oil as the sole carbon source, and 25% of the isolates were capable of growing in the employed conditions. *A. terreus* MUT 271 was the most efficient strain in ONR7a medium with 1% w/v crude oil and 0.1% Tween 80 (24°C, 110 rpm, 10 days), decreasing 40% of the hydrocarbon compounds determined by gas chromatography–mass spectrometry (Bovio et al., 2017).

A wide variety of reactions were performed employing marine-derived fungi, including approaches that unified different green methods aiming for cleaner processes, a feature that will guide future studies in biocatalysis for the obtention of new and interesting compounds.

In the exploration of reactions employing whole-cell catalysis, it is important to emphasize the need for an adequate identification of the marine catalyst, including the deposit of these strains in culture collections registered in the World Data Centre for Microorganisms, making the catalysts and their genome available for the scientific community. Moreover, the enzymes involved in the process should also be characterized, promoting a better understanding of the evaluated processes.

For optative marine microorganisms, studies should focus on the comparison between the performance of different strains of the same species obtained from marine and terrestrial environment, generating a better understanding of the genetic modifications promoted by the selective pressure over the approached organism. In addition, experiments with and without high salt concentrations are worthy of being evaluated.

## Isolated Enzymes

Different classes of enzymes from marine microorganisms were studied in reactions with organic compounds, including oxidoreductases, hydrolases, lyases, and ligases. The bacterial reports presented in the literature were organized according to the international classification of enzymes. Moreover, the studies describing applications of fungal enzymes were also summarized.

### Bacterial Enzymes

#### Oxidoreductases

The enzyme class oxidoreductases contains the biocatalysts for oxidation and reduction reactions. These enzymes are widely used to catalyze the hydroxylation of PAHs or the biotransformation of products for the fine or bulk chemical industries. Some of these compounds are known for causing environmental contamination, and environmentally friendly approaches for reduction of these pollutants are trends in Green Chemistry (Haritash and Kaushik, 2009).

The biodegradation/biotransformation of PAHs into less complex products or even mineralization (H_2_O, CO_2_, and CH_4_) depends on pH, temperature, solvents, growth medium, oxygenation, type of microorganisms, and other parameters (Haritash and Kaushik, 2009). For this reason, biotechnological processes have been studied to isolate and apply enzymes, such as oxydoreductases, to bioremediate or biotransform PAHs.

**FIGURE 7:**
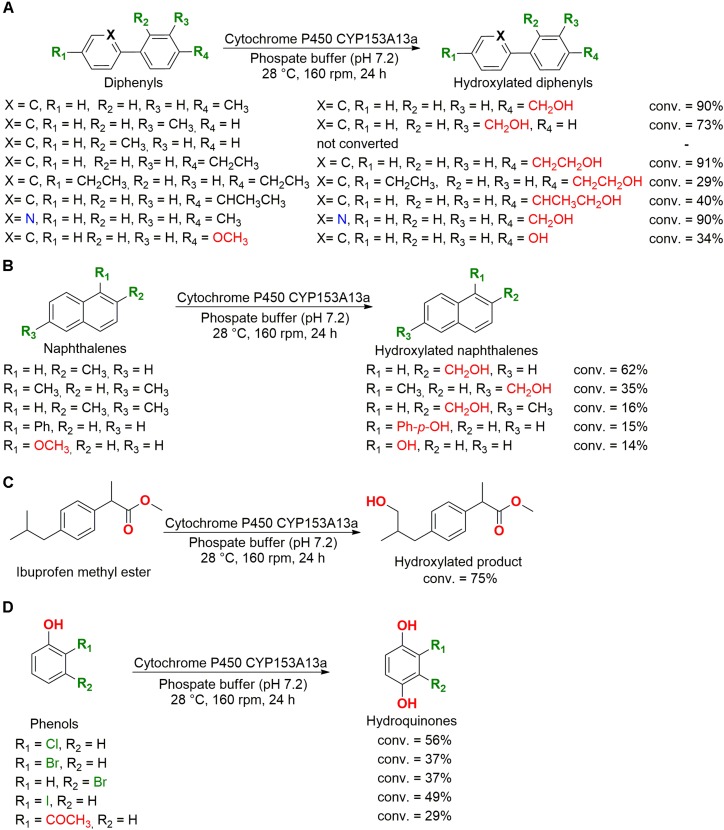
Biotransformation of **(A)** diphenyls, **(B)** naphthalenes, **(C)** ibuprofen methyl ester, and **(D)** phenols by cytochrome P450 CYP153A13a (Otomatsu et al., 2010).

Different monooxygenases and dioxygenases of marine bacteria were studied for application in biocatalysis. For example, the cytochrome P450 CYP153A13a from the bacteria *Alcanivorax borkumensis* was cloned and overexpressed in *Escherichia coli*, showing a potent ability to hydroxylate aromatic compounds with terminal alkyl groups, including diphenyl (Figure 7A), naphthalene (Figure 7B), ibuprofen methyl ester (Figure 7C), and phenol (Figure 7D) derivatives. This cytochrome also hydroxylated the *para* position of halogenated phenolic compounds and demethylated 4-methoxybiphenyl and 1-methoxynaphthalene to hydroxyl compounds (Otomatsu et al., 2010).

**FIGURE 8:**
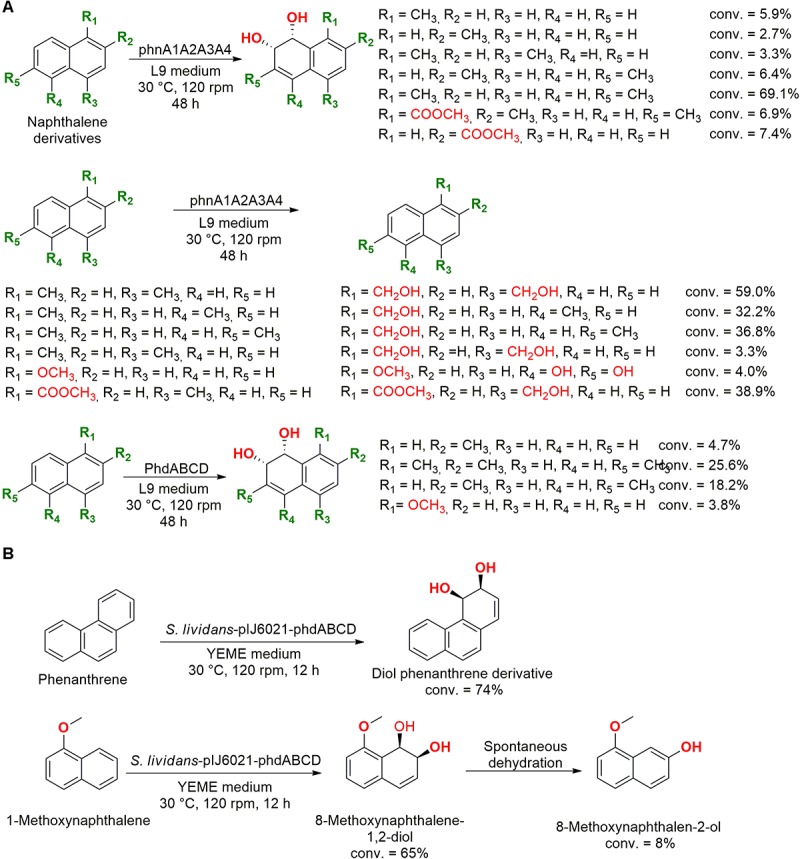
**(A)** Hydroxylation of PAHs using the dioxygenase enzyme phnA1A2A3A4 (Shindo et al., 2007). **(B)** Biotransformation of phenanthrene and 1-methoxynaphthalene into the corresponding *cis*-diol (Chun et al., 2001).

Naphthalene derivatives were also employed as substrate in experiments with two genes encoding the production of dioxygenases isolated from the marine bacteria *Nocardioides* sp. KP7 (phnA1A2A3A4) and *Cycloclasticus* sp. A5 (phdABCD). These enzymes were expressed in *E. coli* and the dioxygenase phnA1A2A3A4 hydroxylated both aromatic rings and/or terminal alkyl groups of the employed substrates (Figure 8A). However, the phdABCD enzyme presented specific hydroxylations only for four naphthalene derivatives (Shindo et al., 2007).

The same gene initially isolated from the marine bacteria *Nocardioides* sp. strain KP7 was genetically modified and introduced into the soil bacteria *Streptomyces lividans* to biotransform phenanthrene and 1-methoxynaphthalene (Figure 8B). The hybrid enzyme (pIJ6021-phdABCD) was capable of dihydroxylating phenanthrene into *cis*-diol phenanthrene and hydroxylate 1-methoxynaphthalene into 8-methoxynaphthalen-2-ol (Chun et al., 2001).

**FIGURE 9:**
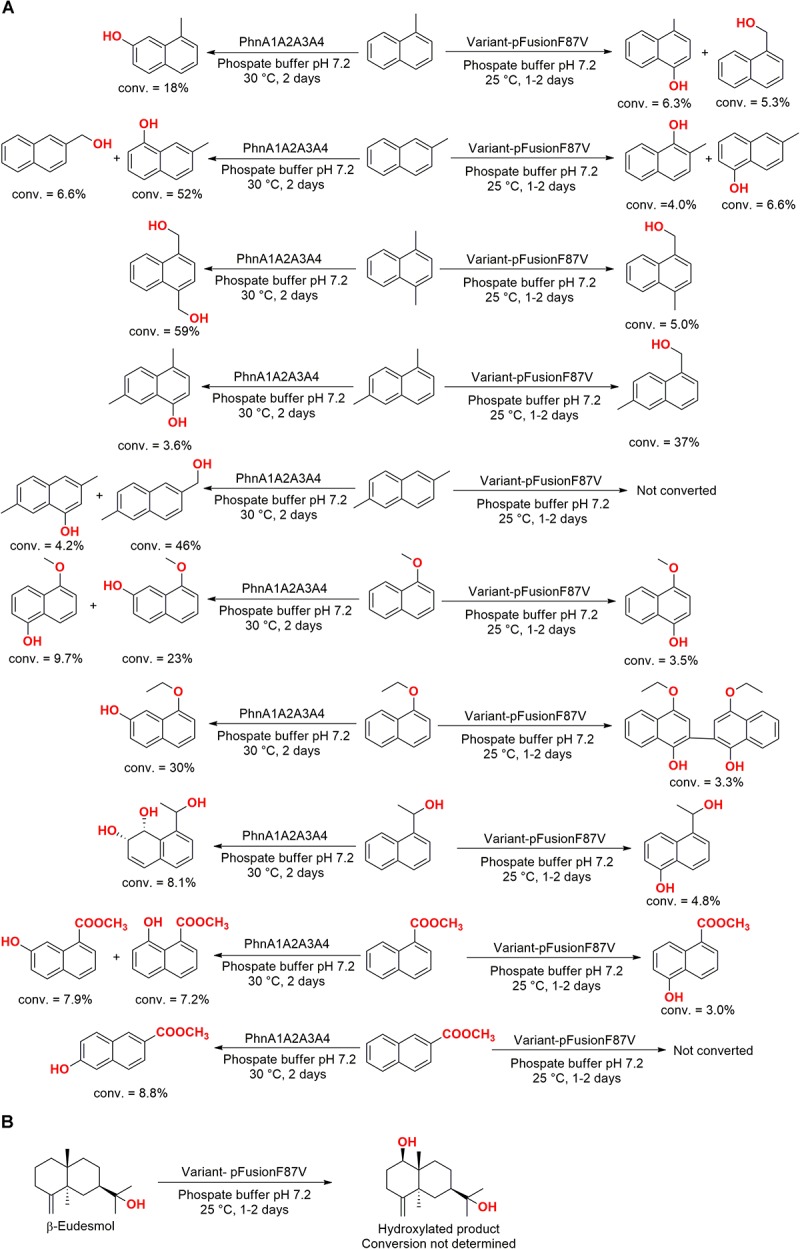
Hydroxylation of **(A)** naphthalene analogs and **(B)** β-eudesmol by the enzymes PhnA1A2A3A4 and pFusionF78V overexpressed in *E. coli* (Misawa et al., 2011).

The enzyme PhnA1A2A3A4 was also employed in a subsequent study with a variant enzyme (pFusionF87V) obtained *via* the genetic manipulation and fusion of the cytochrome P450 BM3 gene with the isomerase gene F87V. These enzymes converted a series of naphthalene analogs (Figure 9A) and β-eudesmol (Figure 9B) to their respective hydroxylated compounds (Misawa et al., 2011).

**FIGURE 10:**
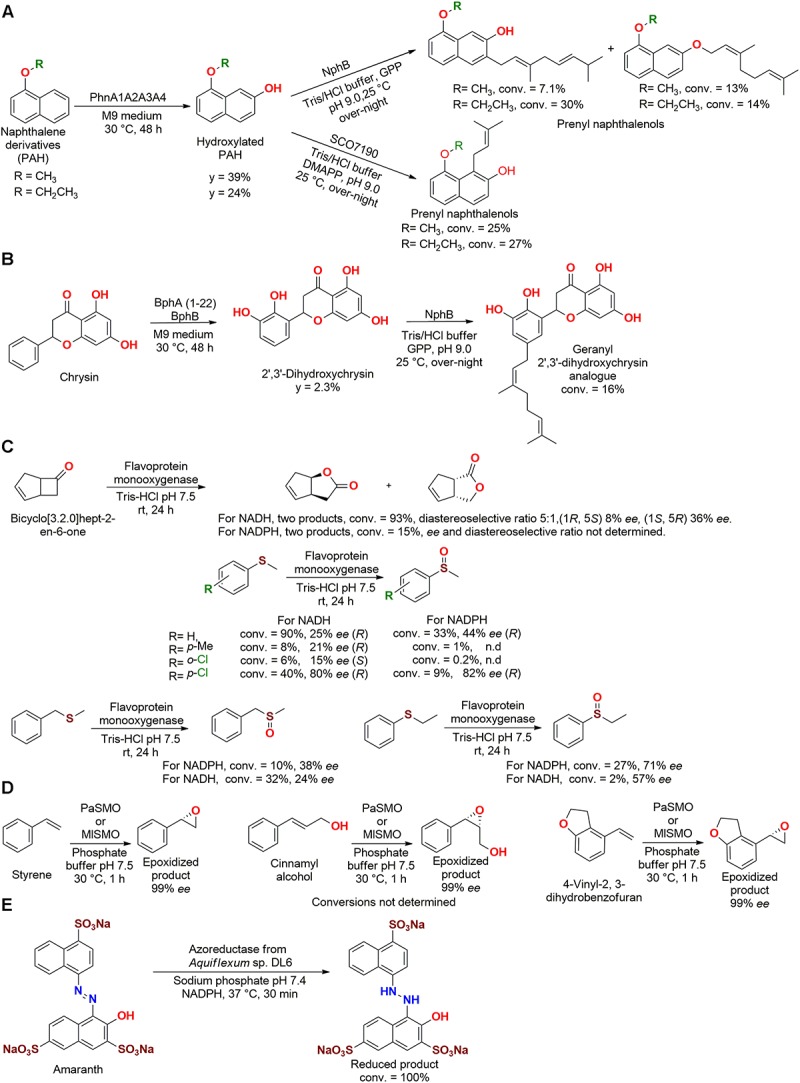
Biotransformation of **(A)** naphthalene derivatives and **(B)** Chrysin by dioxygenase and prenyltransferase enzymes (Shindo et al., 2011). **(C)** Baeyer–Villiger oxidation and thioester oxidation by a flavoprotein monooxygenase (Jensen et al., 2012). **(D)** Epoxidation of styrene derivatives by two monooxygenases isolated from the genome of *P. agarilytica* NO2 and *Marinobacterium litorale* DSM 23545 (Pu et al., 2018). **(E)** Decolorization of Amaranth by an azoreductase enzyme from *Aquiflexum* sp. DL6 (Misal et al., 2013).

Shindo et al. (2011) also employed a combination of enzymes expressed in *E. coli* to produce prenyl naphthalen-ols (Figure 10A) and a geranyl 2′,3′-dihydroxychrysin analog (Figure 10B). The first step was performed by the previous discussed dioxygenase PhnA1A2A3A4 (*Cycloclasticus* sp. A5) that produced hydroxylated PAHs, which were further reacted with geranyl diphosphate (GPP) by the prenyltransferase NphB to produce PHA-GPP products.

Subsequently, a reaction between the hydroxylated PAHs with dimethylallyl diphosphate (DMAPP) produced PHA-DMAPP compounds, which were obtained by the catalysis employing the prenyltransferase SCO7190 from *Streptomyces coelicolor* A3. The flavonoid chrysin was also dihydroxylated by the genetic modified dioxygenase enzymes BphA (1–22) and/or BphB, and was subsequently used to produce the geranyl 2′,3′-dihydroxychrysin analog. The biotransformed compounds demonstrated an improved antioxidant activity (IC_50_ = 5 to >100 μM) in lipid peroxidation of rat brain, when compared to the precursor substrates (Shindo et al., 2011).

A different alkane hydroxylase (AlkBI) obtained from the marine bacteria *A. borkumensis* SK2 was studied for its potential to hydroxylate alkanes with 5–12 carbons, as observed by Smits et al. (2003) using a similar enzyme from *Pseudomonas aeroginosa* PAO1. Cloned and expressed in *E. coli*, the purified monooxygenase from *A. borkumensis* SK2 was dependent of the cofactor NADPH, which was recycled using rubredoxin and a rubredoxin reductase enzyme (Eidani et al., 2012).

Other types of oxidation reactions, Baeyer–Villiger oxidation and thioester oxidation, were carried out by a flavoprotein monooxygenase obtained from the marine bacteria *Stenotrophomonas maltophilia* genome, which was cloned and expressed in *E. coli* (Figure 10C). The enzyme was active with both NADH and NADPH cofactors, but the Baeyer–Villiger oxidation of bicyclo[3.2.0]hept-2-en-6-one presented better conversions using NADH (*K*_M_ = 23.7 μM, *k*_cat_ = 0.029 s^–1^) than NADPH, as also observed for thioesters (Jensen et al., 2012).

In a study aiming for organic synthesis, styrene monooxygenases identified in the genome of *Paraglaciecola agarilytica* NO2 and *Marinobacterium litorale* DSM 23545 were expressed in *E. coli.* Both crude enzymes (PaSMO and MISMO, respectively) presented optimum activity at 30°C and pH 8. These biocatalysts carried out the epoxidation of styrene derivatives (styrene, cinnamyl alcohol, 1-phenylprop-2-en-1-ol, and 4-vinyl-2, 3-dihydrobenzofuran), producing the *S*-enantiomer up to 99% *ee* (Figure 10D) (Pu et al., 2018).

In an interesting study, the red marine bacteria *Aquiflexum* sp. DL6 produced an enzyme with azoreductase and nitroreductase activity. The isolated enzyme was NADPH dependent with an optimum pH and temperature of 7.4 and 60°C, respectively. After being tested with 14 azo dyes, the enzyme reduced Amaranth with 100% conversion (*K*_m =_ 1.11 mM and *V*_max_ = 30.77 U/mg) (Figure 10E). Other dyes such as Fast red E (87%), Sunset yellow FCF (66% conversion), and some nitro-compounds (2-nitro aniline, 66% conversion; 4-nitro phenol, 69% conversion; 3-nitro benzaldehyde, 78% conversion; 1-chloro 2-nitro benzene, 76% conversion; 4-nitro benzoic acid, 81% conversion) were also reduced with good efficiency under the same conditions. However, the produced compounds were not characterized (Misal et al., 2013).

Oxidoreductases from different marine bacteria were studied for different applications, including the functionalization of aliphatic and aromatic compounds for the synthesis of more complex substances and the decolorization of dyes, showing the potential of the described enzymes for these applications.

#### Hydrolases

The hydrolases are the most applied class of enzymes in bio-chemistry and industrial processes. The main reasons for this increased number of applications are the enzymatic stability and the lack of need of cofactors, which are usually expensive. Hydrolases can perform the hydrolytic cleavage of C–O, C–N, and C–C bonds in water media and the formation of these same bonds in organic solvents. Enzymes such as lipases and esterases are also employed to carry out kinetic resolutions for production of chiral building blocks or compounds with biological potential (Mane, 2016).

**FIGURE 11:**
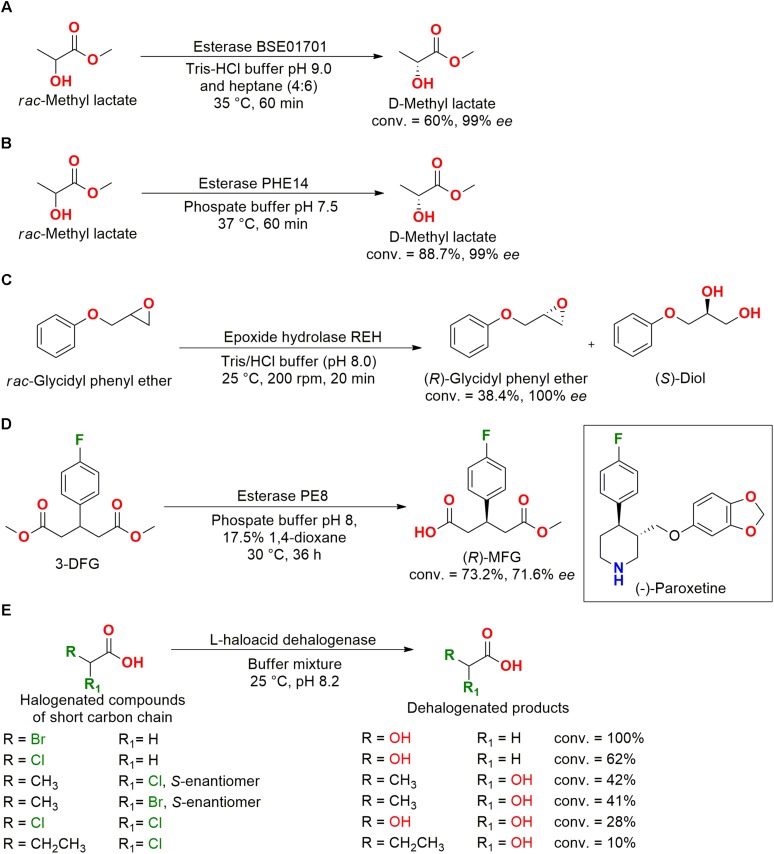
Resolution for D-methyl lactate production by **(A)** esterase BSE01701 (Huang et al., 2016) and by **(B)** esterase PHE14 (Wang Y.L. et al., 2016). **(C)** Kinetic resolution of *rac*-glycidyl phenyl ether into the (*R*)-enantiomer by the epoxide hydrolase REH (Woo et al., 2010). **(D)** Production of (*R*)-3MFG, a precursor for (–)-paroxetine hydrochloride drug synthesis, employing the esterase PE8 (Wei et al., 2013). **(E)** Dehalogenation of monobromated carboxylic acids by the L-haloacid dehalogenase HADII_BSW_ from *Pseudoalteromonas* sp. BSW20308 (Liao et al., 2015).

Some hydrolases from marine bacteria were studied and applied to the kinetic resolution of different compounds, for example, the kinetic resolution of racemic methyl lactate performed by the esterase BSE01701, which was cloned and isolated from the deep-sea *Bacillus* sp. SCSIO 15121. This enzyme was resistant to different surfactants and organic solvents, producing the D-methyl lactate in great enantiomeric excess (99% *ee*) with interesting conversion and time (Huang et al., 2016) (Figure 11A).

Another esterase, PHE14, was also cloned from deep-sea bacteria (*Pseudomonas oryzihabitans* HUP022) and presented good activity in resolving racemic methyl lactate. PHE14 was resistant to organic solvents, surfactants, metal ions, and high NaCl concentrations (Figure 11B). The D-lactic acid and its ester analogs present a critical role as building blocks for drug synthesis and fine chemicals (Huang et al., 2016; Wang Y.L. et al., 2016).

In another example of hydrolysis reaction, enantiopure (*R*)-glycidyl phenyl ether was obtained using an epoxide hydrolase identified from the *Maritimibacter alkaliphilus* KCCM 42376 genome. The cloned gene, which was overexpressed in *E. coli*, enabled the purification of the enzyme REH that preferentially hydrolyzed the (*S*)-glycidyl phenyl ether forming the diol analog (Figure 11C). The enantiospecific hydrolysis was interesting for the obtention of enantiopure epoxides and diols (Woo et al., 2010).

The esterase PE8 cloned and isolated from the marine bacteria *Pelagibacterium halotolerans* B2^T^ and characterized as an alkaline enzyme with a good enantioselectivity for the desymmetrization by hydrolysis of the dimethyl 3-(4-fluorophenyl)glutarate (3-DFG) into the chiral compound methyl (*R*)-3-(4-fluorophenyl)glutarate (*R*)-3MFG (Figure 11D). (*R*)-3MFG is considered an important precursor for the synthesis of (–)-paroxetine hydrochloride, a drug commonly used as antidepressant (Wei et al., 2013).

Different studies were carried out approaching the ester hydrolysis on model substrates such as *p*-nitrophenyl butyrate and other *p*-nitrophenyl esters. For EstH, cloned and purified from the marine bacteria *Zunongwangia* sp., an improvement of the esterase activity was performed by enzyme immobilization on a nano-composite of Fe_3_O_4_∼ cellulose. Using *p*-nitrophenyl butyrate, the free enzyme presented optimal conditions at 30°C and pH 8.5 with *V*_max_ = 35.76 μM/min, *k*_cat_ = 365 s, 16 h of half-life, 40% of storage stability after 50 days, 22.40% of temperature stability at 50°C, high NaCl tolerance (0–4.5 M), organic solvent, and surfactant stability (Rahman et al., 2016).

After being immobilized, the optimal temperature increased to 35°C with a wider pH stability range of 4–10, *V*_max_ = 51.14 μM/min, *k*_cat_ = 520 s, 32 h of half-life, 71% of storage stability after 50 days, and 48.5% of temperature stability at 50°C. The esterase EstH could also be recycled eight times until a reduction of its activity to 50%, suggesting the great potential of this biocatalyst for biotechnological processes (Rahman et al., 2016).

Another esterase gene was cloned from the marine bacteria *Erythrobacter seohaensis* SW-135 and expressed in *E. coli* encoded a 274-amino-acid protein. The enzyme activity characterization was performed using *p*-nitrophenyl esters (C2–C16), and the best result was obtained toward *p*-nitrophenyl butyrate (16.0 ± 1.1 U/mg). The optimum temperature (60°C), pH (10.5), and tolerance to NaCl (up to 3 M), organic solvent, and Triton X-100 were determined and considered interesting, placing this esterase as a promising biocatalyst for industrial applications (Huo et al., 2017).

Esterases were also used to biodegrade pesticides; an interesting example was the bioremediation of cypermethrin using the soil bacteria *B. subtilis* 1D, a producer of laccase and esterase enzymes. Under the optimized condition, 95% of the pyrethroid was degraded into less dangerous compounds after 15 days of reaction, showing the potential of this bacteria in environmental decontamination (Gangola et al., 2018).

A lipase isolated from the marine bacteria *Bacillus pumilus* B106, which was associated to a *Halichondria rugosa* sponge, was also studied. This enzyme was characterized by Zhang et al. (2009) through gene cloning and expression in *E. coli*. An increase of 3.54-fold of the lipase activity employing *p*-nitrophenylacetate and 1.31-fold of the cell concentration was observed after improving the medium composition for fermentation, and determining the optimal pH of 8.0 and temperature of 40°C. It is also noteworthy that the use of 10–20% of methanol improved the enzymatic activity, whereas 30–40% of methanol, ethanol, 2-propanol, and DMSO deactivated the enzyme.

Proteases from marine bacteria were also present in the literature. An alkaline protease from a *Bacillus cereus* obtained from crude oil at the Gulf of Khambhat (India) presented tolerance to organic solvents, detergents (Triton X-100 and Tween 80), and oxidizing ions. After purification, the enzyme showed activity for casein hydrolysis with optimal conditions of temperature at 60°C and pH 8.0. Ions such as Li^+^, K^+^, Mg^2+^, and Ba^2+^ did not affect the enzyme activity, unlike the improving effect of Ca^2+^ and the deactivating or reducing action of the ions Cr^3+^, Zn^2+^, Cu^2+^, Hg^2+^, Pb^2+^, and Cd^2+^ (Shah et al., 2010).

Another alkaline protease was isolated from the marine bacteria *Staphylococcus saprophyticus* BUU1 and presented a broad range of temperature (10–80°C) and pH (3-12) for the hydrolysis of casein. The determined kinetic parameters were a *K*_m_ of 0.83 mg/mL and a *V*_max_ of 592.86 mmol/min. This enzyme was also tested in the presence of inhibitors such as metal ions, surfactants, oxidizing agents, bleaching agents, and apolar solvents, and its stability supports future industrial applications due to its resistance (Uttatree and Charoenpanich, 2018).

The marine environment presents high concentrations of halogenic compounds and ions. Therefore, the L-haloacid dehalogenase PinHAD was identified and cloned from the marine bacteria *Psychromonas ingrahamii* DSMZ 17664 isolated from Elson Lagoon (Alaska, United States), showing good activity when applied to halogenated compounds with short carbon chains (≤C3). This enzyme was capable of dehalogenating monobromoacetic acid with 100% conversion (*V*_max_ = 0.6 μM min^–1^ mg^–1^ and *K*_m_ = 1.36 mM) and monochloroacetic acid with 62% conversion (Novak et al., 2013).

An Arctic marine bacteria identified as *Pseudoalteromonas* sp. BSW20308 also presented an L-haloacid dehalogenase (HADII_BSW_) in its genome, which was cloned, expressed, and isolated (Figure 11E). The enzyme showed optimum activity at 40°C in the dehalogenation of monobromoacetic acid with a conversion of 100% (*V*_max_ = 13.22 μM min^–1^ mg^–1^ and *K*_m_ = 0.15 mM). Moreover, (−)-*S*-2-bromopropionic acid was also dehalogenated with a conversion of 41% (Liao et al., 2015).

Polysaccharides are polymers generally produced from renewable sources. These polymers have some important properties, such as biocompatibility, bioadhesivity, biodegradability, non-toxicity, low cost, easy availability, and wide uses in food, biomedical, textile, biofuel, and cosmetic applications (Aravamudhan et al., 2014).

Different types of enzymes from marine bacteria that carry out hydrolysis of polysaccharides have been studied, including chitinase, rhamnosidase, glucuronidase, agarase, xylosidase, xylanase, cellulase, carrageenase, amylase, and fucoidanase. The obtained products from the hydrolysis processes could be applied mainly as food additives or for production of compounds with pharmacological properties.

**FIGURE 12:**
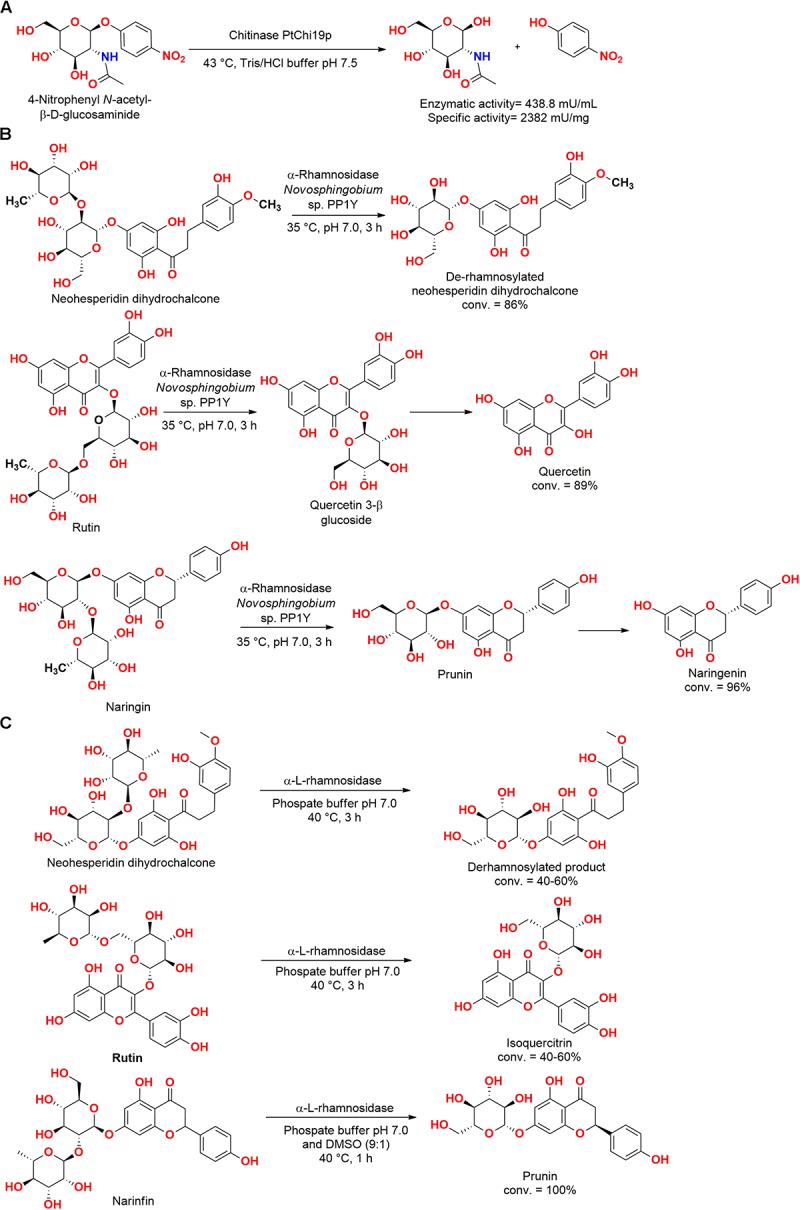
**(A)** Hydrolysis of *p*-nitrophenyl *N*-acetyl-β-D-glucosaminide by the chitinase PtChi19p (Garcia-Fraga et al., 2015). **(B)** Hydrolysis of flavonoid glycosides by an L-rhamnosidase from *Novosphingobium* sp. P1Y (Izzo et al., 2014). **(C)** Production of glycosylated flavonoids by an α-L-rhamnosidase from *Novosphingobium* sp. P1Y (De Lise et al., 2016).

A chitinase gene cloned from the marine bacteria *Pseudoalteromonas tunicata* CCUG 44952T allowed the obtention of the enzyme PtChi19p, which belongs to the chitinase family 19. The chitinase PtChi19p was active in the hydrolysis of crystalline chitin (3 mU/ml, 16 mU/mg), colloidal chitin (11 mU/ml, 60 mU/mg), and *p*-nitrophenyl *N*-acetyl-β-D-glucosaminide (438.8 mU/ml, 2382 mU/mg) (Figure 12A). Moreover, this enzyme presented antifungal activity against *Fusarium oxysporum*, *Armillaria mellea*, *Trichophyton mentagrophytes*, *Microsporum gypseum*, and *Aspergillus niger*, which are well-known pathogenic fungi for plants or humans (Garcia-Fraga et al., 2015).

In an interesting study approaching hydrolysis reactions, a non-purified extract of the bacteria *Novosphingobium* sp. PP1Y isolated from seawater of the harbor of Pozzuoli (Naples, Italy) presented excellent L-rhamnosidase activity, hydrolyzing both 1–2 and 1–6 interglycosidic bonds of flavonoid glycosides (Figure 12B). This biocatalyst was employed in the production of flavonoids, successfully employing naringin, rutin, and neohesperidin dihydrochalcone as substrate (Notomista et al., 2011; Izzo et al., 2014).

In a subsequent study, an α-L-rhamnosidase from *Novosphingobium* sp. PP1Y was obtained by cloning and overexpression in *E. coli*. The α-L-rhamnosidase was stable up to 45°C and pH 6.9, hydrolyzing naringin, rutin, and neohesperidin dihydrochalcone into their respective glycosylated flavonoids, representing a potential valuable enzyme for the biotechnological industry (Figure 12C) (De Lise et al., 2016).

A five-domain α-glucuronidase (SdeAgu115A) isolated from the marine bacteria *Saccharophagus degradans* 2-40^T^, isolated from a decomposing *Spartina alterniflora* at a Chesapeake Bay salt marsh in Matthews County (United States) (Andrykovitch and Marx, 1988), was active in the hydrolysis of beechwood xylan (2470 μmol product/min/μmol enzyme), spruce arabinoglucuronoxylan (917 μmol product/min/μmol enzyme), and oat spelt xylan (24 μmol product/min/μmol enzyme), which are substrates used for sugar conversion. The α-glucuronidase family usually presents a four-domain enzyme structure; the fifth domain identified by Wang W.J. et al. (2016) using X-ray and other spectroscopy analyses revealed that its presence implied a significant impact on monomer arrangement and dimerization, influencing the enzyme activity and stability.

Since there are few studies about conformation and stability of sulfatases, Neira et al. (2016) determined the structural features assumed by the dimer 4-*O*-endosulfatase obtained from the bacteria *Vibrio* sp. FC509, which was isolated from coastal sediment in the Jiaozhou Bay (Qingdao, China) (Han et al., 2014). This enzyme removed the 4-*O*-sulfate from chondroitin sulfate and dermatan sulfate. Moreover, this study presented the optimal pH (7–9) for enzymatic activity, the secondary structure (mixture of α and β sheets), and observed that the dimer was not linked by a disulfide bridge. In addition, thermal and chemical procedures irreversibly denaturized the enzyme but did not completely unfold the structure during heating.

**FIGURE 13:**
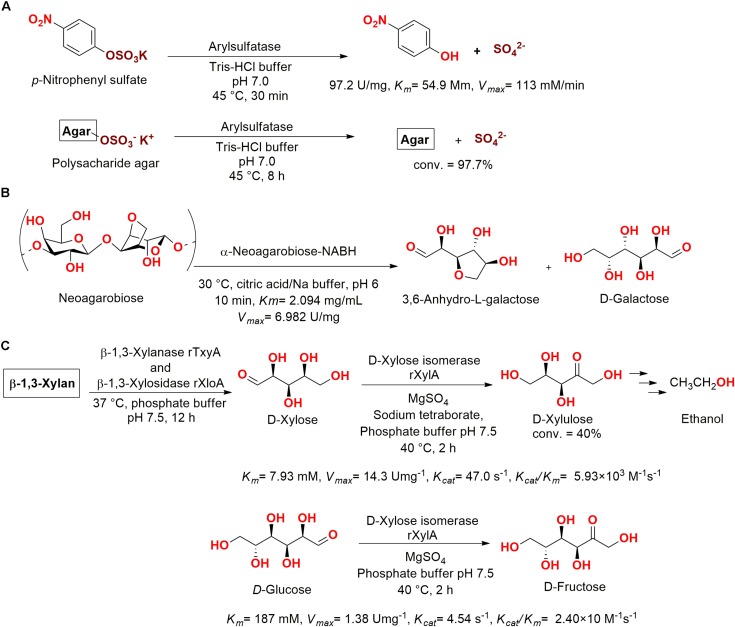
**(A)** Hydrolysis of sulfate group using an arylsulfatase enzyme (Kim et al., 2004). **(B)** Hydrolysis of neoagarobiose into 3,6-anhydro-L-galactose and D-galactose (Liu et al., 2016). **(C)** D-xylulose production from β-1,3-xylan using employing the enzymes β-1,3-xylanase TxyA, β-1,3-xylosidase XloA, and D-xylose isomerase XylA from *Vibrio* sp. XY-214 (Umemoto et al., 2012).

Kim et al. (2004) also characterized an arylsulfatase, which was obtained from the marine-derived bacteria *Sphingomonas* sp. AS6330 isolated from the Southeastern coast of Korea (deposited as KCTC 2817). After purification, the enzyme had a 12,800-fold purity increase (97.2 U/mg) and presented a great activity when used to cleave sulfate ester bonds from agar (97.7% of sulfate bonds cleavage) and *p*-nitrophenyl sulfate (*K*_m_ = 54.9 mM, *V*_max_ = 113 mM/min) (Figure 13A).

An α-neoagarobiose enzyme (361 amino acids, 41 kDa) was cloned from the marine bacteria *Agarivorans gilvus* WH0801 obtained from a seaweed at the coast of Weihai (China) (Du et al., 2011). This enzyme degraded neoagarabiose producing 3,6-anhydro-L-galactose and D-galactose, which are two examples of sugars widely employed in health care and in the food industry (Figure 13B) (Liu et al., 2016).

A β-agarase gene was also identified in the *A. gilvus* WH0801 genome by Liu et al. (2014). This enzyme was cloned and expressed in *E. coli* and, after purification, was capable of hydrolyzing agarose into neoagarotetraose (30°C, citric acid/Na buffer pH 6.0, *K*_m_ = 5.97 mg/mL and *V*_max_ = 0.781 U/mg).

In another study, an isomerase obtained from *Vibrio* sp. XY-214 isolated from sea mud at Ise Bay (Japan) was cloned, expressed in *E. coli*, and identified as the D-xylose isomerase XylA. Aiming for the fermentation of β-1,3-xylan for ethanol production by *Saccharomyces cerevisiae*, the previous reported enzymes β-1,3-xylanase TxyA and β-1,3-xylosidase XloA, which were also obtained from *Vibrio* sp. XY-214, were employed for saccharification of β-1,3-xylan into the sugar D-xylose (*K*_m_ = 7.93 mM). Then, D-xylose was bio-converted using XylA into D-xylulose, which was employed for fermentation with *S. cerevisiae*, providing a basis for ethanol production from β-1,3-xylan (Figure 13C) (Umemoto et al., 2012).

Cellulases are hydrolytic enzymes mostly used for deconstruction of lignocellulosic biomass for production of biofuel and different chemicals. Nowadays, this type of chemistry has received great attention for obtention of second-generation biofuels by the search of new microorganisms and enzymes capable of carrying out this process with robustness (Passos et al., 2018).

A cellulase isolated from the marine bacteria *Bacillus* sp. SR22 obtained from *Siderastrea stellata* colonies at Cabo Branco coral reefs (Brazil) was resistant to variations of salt, temperature, and pH. It is important to note that the enzymes produced by *Bacillus* sp. SR22 were studied because this strain was able to grow with carboxymethylcellulose and sugarcane bagasse as the sole carbon source. This cellulase was identified as an *endo*-β-1,4-glucanase with optimal conditions at pH 6.5 and 60°C, maintaining more than 80% of its residual activity after 1 h of incubation in 1.5 M NaCl aqueous solution. The kinetic parameters were 0.704 mg mL^–L^ (*K*_m_) and 29.85 μmol mL^–l^ min^–1^ (*V*_max_) (dos Santos et al., 2018).

Soil bacteria obtained from oak forest were also employed for the biodegradation of cellulose and hemicellulose. The bacteria *Pedobacter* O48 and *Mucilaginibacter* L294 showed activity to decompose these and other polysaccharides present in wood samples (Lopez-Mondejar et al., 2016). The soil bacteria *B. cereus*, *B. subtilis*, and *Bacillus thuringiensis* from 57 isolates were the only strains able to hydrolyze cellulose, reaching a maximum activity of 0.440 IU/ml/min (Patagundi et al., 2014).

A ι-carrageenase gene was cloned from the bacteria *Wenyingzhuangia fucanilytica* CZ1127^T^ isolated from a seawater sample at Jiaozhou Bay (China) (Chen et al., 2016) and expressed in *E. coli.* The enzyme optimum activity was achieved at 25°C and pH 8. In addition, characterized as an *endo*-acting hydrolase, this enzyme produced as major products tetrasaccharides, disaccharides, and hexasaccharides, respectively. The kinetic parameters obtained using ι-carrageenan as substrate were 1.12 μM (*K*_m_) and 560.75 s^–1^ (*k*_cat_) (Shen et al., 2017).

Amylases are one of the main hydrolases used in the food industry. This sub-class of enzymes performs the hydrolysis of starch for production of glucose units, which are largely used as food sweeteners (De Souza and Magalhaes, 2010). However, studies should be carried out to isolate and determine the optimal experimental conditions for enzymatic catalysis of this reaction for the achievement of greater results.

An alkaline and organic solvent-tolerant α-amylase from *Catenovulum* sp. X3 isolated from seawater of the coastal sea of Shantou (China) was expressed and cloned in *E. coli*. After purification, this enzyme was applied to biohydrogen production by starch saccharification, and was also able to hydrolase amylopectin and amylose. The performed hydrogen production by starch fermentation was 3.73-fold higher with the amylase treatment than without the enzyme use. The optimum enzyme activity was observed at 35°C and pH 9.0 with maltose as the main oligosaccharide obtained from the hydrolysis reaction (Wu et al., 2017).

In a more recent work, a recombinant fucoidanase from the marine bacteria *Formosa algae* KMM 3553^T^ isolated from the brown algae *Fucus evanescens* (Kraternaya Bay, Russia) (Ivanova et al., 2004) was applied to the hydrolysis of fucoidan into different derivatives. The polysaccharide was mainly hydrolyzed into tetrasaccharides (45% of the obtained products) and a polysaccharide fraction of high-molecular-weight products (HMP, 55% of the obtained products). The fucoidan and HMP were able to inhibit the growth of colon carcinoma cells DLD-1, HCT-116, and HT-29. Moreover, HMP presented a better cytotoxic activity than the native fucoidan (Silchenko et al., 2013, 2018).

Therefore, hydrolases from marine bacteria were applied for different purposes in biotechnological processes, showing the potential of marine enzymes for the chemical industry from different sectors, including pharmaceutical, food, and biomass processing.

#### Lyases

The lyases are a class of enzymes that cleave C–C, C–O, C–N, and other bonds, without performing a hydrolysis or an oxidation reaction. These biocatalysts catalyze very important transformations, and their activity and stability should be evaluated (McDonald et al., 2015).

**FIGURE 14:**
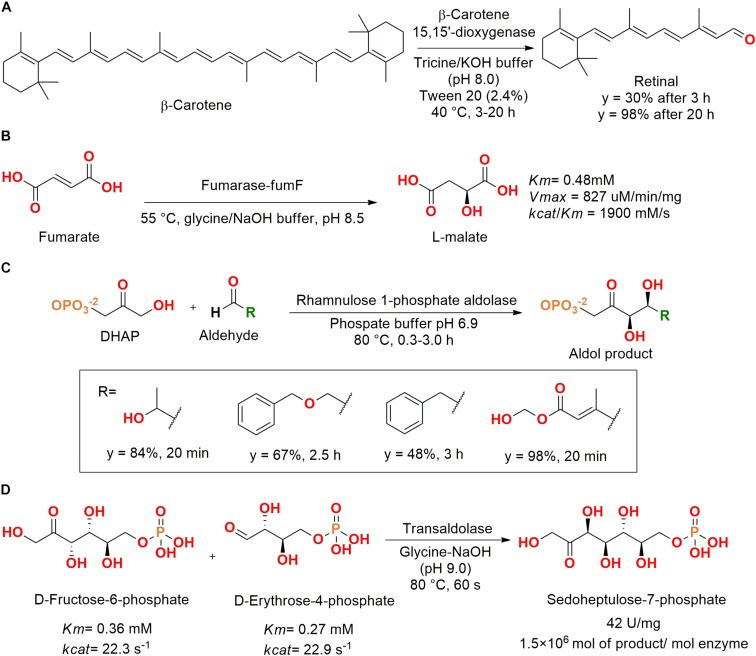
**(A)** Production of retinal from β-carotene using a β-carotene 15,15′-dioxygenase (Kim et al., 2010). **(B)** Obtention of L-malate by hydration of fumarate catalyzed by a fumarase enzyme (Jiang et al., 2010). **(C)** Aldol reaction by a thermophilic aldolase isolated from *T. maritima* MSB8 (Oroz-Guinea et al., 2015). **(D)** Production of sedoheptulose-7-phosphate by a thermophilic transaldolase isolated from *T. maritima* MSB8 (Huang et al., 2012).

Lyases from marine bacteria were assessed. For example, retinal was produced from β-carotene using a recombinant β-carotene 15,15′-dioxygenase enzyme from an unculturable strain (GenBank accession number AAY68319), which was cloned in *E. coli*. It is important to note that to dissolve the β-carotene, detergent micelles were produced using toluene as organic solvent and Tween 20, allowing the solubilization of β-carotene in tricine/KOH buffer and the enzymatic catalysis for retinal production (Figure 14A) (Kim et al., 2010).

In another study, researches employed a metagenomic library screening for obtention of a fumarase from an unculturable marine bacteria. The fumarase fumF gene was subcloned into a pETBlue-2 vector, which was expressed in *E. coli* and purified. After purification, the enzyme was active for the catalysis of fumarate hydration into L-malate, showing optimal activity at 55°C, pH 8.5, and 5 mM of Mg^2+^ (Figure 14B) (Jiang et al., 2010).

Aiming for extreme enzymes, a thermophilic gene from *Thermotoga maritima* MSB8 isolated from anaerobic marine mud at the Vulcano island (Italy) was identified as a rhamnulose 1-phosphate aldolase. After cloning and expression in *E. coli*, the purified enzyme showed optimal activity at 95°C and was activated by Co^2+^ instead of Zn^2+^, as usually observed for *E. coli* (Nelson et al., 2001; Oroz-Guinea et al., 2015).

The enzyme also retained 90% of its activity in the presence of acetonitrile (40%) and maintained 50% of the initial activity until 3 h at 115°C. The kinetic parameters for rhamnulose-1-phosphate were determined as *K*_m_ = 3.6 mM and *k*_cat_*/K*_m_ = 0.7 × 10^3^ s^–0^ M^–0^ at room temperature. The enzyme also catalyzed the aldol reaction between dihydroxyacetone phosphate (DHAP) and four different aldehydes in good conversions and times (Figure 14C) (Oroz-Guinea et al., 2015).

Another hyper-thermophilic transaldolase was also identified in *T. maritima* MSB8 genome and obtained by gene cloning and expression in *E. coli*. The transaldolase TAL was able to catalyze the aldol reaction between D-fructose-6-phosphate and D-erythrose-4-phosphate to produce sedoheptulose-7-phosphate, which is an important precursor for compounds with biological applications, biofuels, and carbohydrates (Figure 14D). This enzyme also maintained up to 50% of its activity until 198 h at 60°C and 13 h at 80°C (Huang et al., 2012).

**FIGURE 15:**
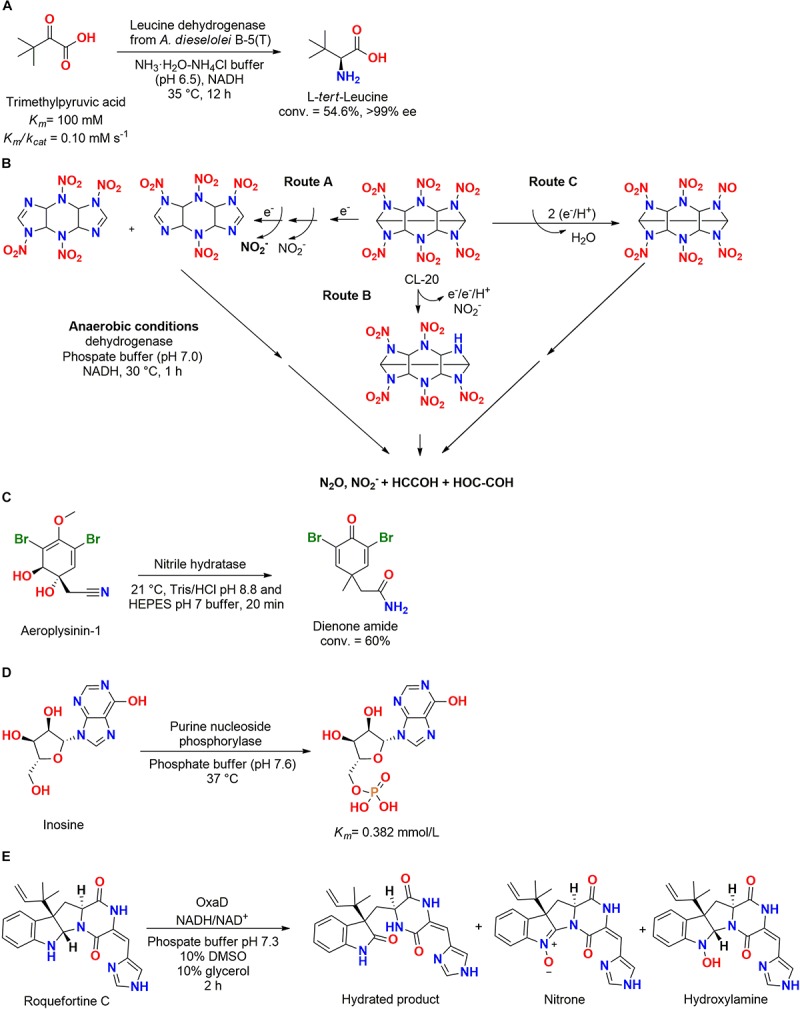
**(A)** Biotransformation of trimethylpyruvic acid in L-*tert*-leucine by a leucine dehydrogenase from *A. dieselolei* B–5(T) (Jiang et al., 2016). **(B)** Biotransformation of CL-50 by a dehydrogenase obtained from *Clostridium* sp. EDB2 (Bhushan et al., 2005). **(C)** Biotransformation of aeroplysinin-1 by a nitrile hydratase into a dienone amide antibiotic (Lipowicz et al., 2013). **(D)** Phosphorylation of the nucleotide inosine by purine nucleoside phosphorylase enzyme from *Pseudoalteromonas* sp. XM2107 (Wang et al., 2010). **(E)** The use of the flavin-dependent nitrone synthase OxaD from the fungus *P. oxalicum* F30 for roquefortine C biotransformation (Newmister et al., 2016).

A extremophilic leucine dehydrogenase gene from *Alcanivorax dieselolei* B-5(T) (Marine Culture Collection of China 1A02288) isolated from surface seawater at Bohai Sea (China) (Liu and Shao, 2005) was cloned and expressed in *E. coli*, presenting a specific activity of 0.88 U/mg and affinity to catalyze reactions in cold temperatures, 0–37°C (Figure 15A). Trimethylpyruvic acid was employed as substrate and the leucine dehydrogenase enzyme produced L-*tert*-leucine, which is an important construction block for chiral drugs (Jiang et al., 2016).

Another dehydrogenase enzyme was used for degradation of CL-20, a cyclic nitramine explosive. The employed enzyme was obtained from the bacteria *Clostridium* sp. EDB2 isolated from marine sediment at the Halifax harbor (Canada). In an anaerobic system, four identified metabolites produced after 15 min of reaction (18.5 nmol of CL-20 per mg of protein) allowed the proposition of at least three different pathways for biodegradation (Figure 15B) (Bhushan et al., 2004, 2005).

In another biotransformation study, a specific nitrile hydratase isolated from the marine sponge *Aplysina cavernicola* or one of its symbiotic microorganisms obtained at the island of Elba (Italy) biotransformed aeroplysinin-1 (AERO) into the dienone amide verongiaquinol (DAV) (Figure 15C). These two compounds (AERO and DAV) presented strong antibiotic activity and were produced as a defense response to predators using brominated isoxazoline alkaloids as precursors. The enzyme was highly specific since it did not catalyze reactions with AERO analogs with small differences at the structure (Lipowicz et al., 2013).

A different type of enzyme, a carbonic anhydrase (CA) able to hydrate CO_2_, was also obtained from the marine bacteria *Hydrogenovibrio marinus* DSM 11271 isolated from seawater at Shonan Coast (Japan). The enzymatic structure presented an unusual *N*-terminal extension when compared to mesophilic bovine CA; this difference probably improved the enzyme solubility in solvents, stability to anionic inhibitors (NO_3_^–^, NO_2_^–^, and Cl^–^), and halotolerance. Therefore, this enzyme could be a potent biocatalyst to capture and utilize CO_2_ in high-salt environments (Nishihara et al., 1991; Jo et al., 2018).

Studies involving alginate lyases were performed, and a gene from the marine bacterium *Vibrio* sp. NJU-03 was cloned and expressed in *E. coli*. The alginate lyase AlgNJU-03 was versatile in the catalysis of poly α-L-guluronate (*K*_m =_ 4.00 mM), alginate (*K*_m_ = 8.50 mM), and poly β-D-mannuronate (*K*_m_ = 10.94 mM), releasing disaccharides, trisaccharides, and tetrasaccharides as major products. The optimum pH and temperature were 7.0 and 30°C, respectively, which is an interesting condition to produce oligosaccharides with low polymerization degree (Zhu B.W. et al., 2018).

Another alginate lyase was obtained from the marine bacteria *Vibrio furnissii* H1 isolated from rotten seaweed at Lianyungang City (China). This enzyme was purified and characterized as a 35.8-kDa protein with 2.40 U/mg of enzyme activity and 7.5 and 40°C of optimal pH and temperature, respectively. The kinetic parameters using sodium alginate as substrate were 2.28 mg/mL (*K*_m_) and 2.81 U/mg (*V*_max_), producing di-, tri-, and tetra-alginate oligosaccharides. Moreover, this enzyme was able to degrade polyguluronate and polymannuronate polysaccharides (Zhu X.Y. et al., 2018).

In summary, marine lyases were employed for different purposes, including the production of different building blocks, oligosaccharides, and biotransformation and biodegradation reactions.

#### Ligases

Ligases from marine microorganisms were poorly studied, and for our knowledge, just one enzyme was reported in the literature. A purine nucleoside phosphorylase was obtained from the marine bacteria *Pseudoalteromonas* sp. XM2107 genome and was active in the phosphorylation of the nucleotide inosine (Figure 15D) (Wang et al., 2010). Therefore, this class of enzymes should be better explored.

The International Union of Biochemistry and Molecular Biology (IUBMB) classified the enzymes in six groups (oxidoreductases, transferases, hydrolases, lyases, isomerases, and ligases). As observed in this review, four of these classes (oxidoreductases, hydrolases, lyases, and ligases) were studied, and isolated enzymes were applied in biotechnological applications, or had its stability assessed under different solvents, media, pH, temperature, and other conditions. These studies are very important to enrich the development of Green Chemistry, replacing processes based on conventional chemistry and oil derivatives for renewable resources and sustainable technologies.

### Fungal Enzymes

Few examples of marine enzymes isolated from fungi have been reported in the literature. This can be due to the difficulty, when compared to bacterial strains, for adaptation of these microorganisms to differing conditions from the marine environment. The period of growth and obstacles for large-scale fermentation could also contribute to the reduced number of studies using fungi and other microorganisms such as algae, sponges, and yeasts. In addition to these challenges, there are few groups studying biotechnological processes by marine fungi, offering an opportunity to researchers in this area.

An interesting study presented a statistical optimization of the production of an agarase using a semi-solid fermentation process and *Palisada perforata* as basal substrate. This agarase was obtained from the fungus *Dendryphiella arenaria* isolated from the red alga *P. perforata* (Red Sea, Egypt). The optimum activity was 7.69 U ml^–l^, and the saccharification of seaweed using crude agarase produced 26.15 mg g^−1^ of sugars (Gomaa et al., 2017).

Another statistical optimization was performed by Gomaa et al. (2018) for the saccharification of the polysaccharides fucoidan and alginate from the brown macroalgae *Sargassum latifolium* using the enzymes fucoidanase and alginate lyase from the same fungus *D. arenaria*. The best activity was 24 U ml^–l^, providing the reduction of 365 mg g^–g^ of fucoidan and 439.66 mg g^–g^ of alginate. These results represent a great achievement for the production of biofuels from marine seaweed.

A fascinating study approached the enzyme OxaD, a flavin-dependent nitrone synthase that was cloned from the fungus *Penicillium oxalicum* F30 genome (GenBank: KX601657) and expressed in *E. coli*. Both cofactors NADH and NADPH were able to biotransform roquefortine C into different products (nitrone, hydrated product, MeOH adduct, and hydroxylamine derivatives) (Figure 15E). Moreover, the compound notoamide S was also transformed by the enzyme OxaD into a different compound though a 2,3-indole epoxidation reaction (Newmister et al., 2016).

The reduced number of studied enzymes from fungi shows that the extensive potential of the sea is still unknown and poorly explored for biocatalysis, constituting an opportunity for researches. Moreover, enzymes from marine bacteria and fungi should be evaluated in terms of reactional yield, enantiomeric excess, and productivity, aiming for pilot testing and large-scale applications. Otherwise, these marine biocatalysts will remain forgotten in the literature.

## Conclusion and Perspective

Bacteria and fungi have been used as whole-cell biocatalysts in different biotechnological processes, including reactions aiming for specific products and the development of biotransformation/biodegradation reactions. The screening of different strains for a desired reaction in whole-cell processes was an important approach for the obtention of interesting catalysts, which enabled the production of different compounds with simplified procedures and reduced costs. However, more sophisticated approaches employing the cloning and expression of enzymes were explored in the recent literature.

A significant number of studies were reported aiming for the identification, isolation, and production of bacterial enzymes; however, few studies of enzymes from fungi were presented, although studies with whole cells of fungi showed the potential of these eukaryotic microorganisms for biocatalysis. Probably, this reduced number of investigations is related to the decreased number of research groups in this area, when compared with bacterial enzymes. In addition, it is noteworthy that these strains must be correctly isolated, identified, and deposited in registered libraries, making their genomes available.

Comparisons between microorganisms from different environments might also be performed, including optative marine species, which also present terrestrial strains that can be studied. Moreover, the presented biocatalytic methods should be evaluated in terms of productivity and enantioselectivity for development of large-scale processes, turning into reality the potential of these marine catalysts.

Although analytical techniques for compound identification have greatly advanced, many studies involving organic compounds employing catalysis by whole-cells or enzymes did not present the characterization of the obtained products. Unfortunately, this is probably a result of purification difficulties for the obtention of materials from biotechnological processes often performed on small scale.

Future biotechnological studies and process development should rely on molecular biology for the obtention of enzymes with unique natural properties, as that reported for marine microorganisms, but with improved efficiency provided by directed evolution. Researchers should also focus on the elucidation of the enzymatic mechanisms of reaction and the expansion of the substrate scope of the already presented enzymes, which would promote the discovery of new applications of the studied biocatalysts.

## Author Contributions

WB was responsible for summarizing 50% of the approached papers, generating a full text with all the authors contributions, organizing, reviewing, and submitting the manuscript. RL summarized the other 50% of the approached literature. AP overviewed the writing process and revised the manuscript with corrections and suggestions.

## Conflict of Interest Statement

The authors declare that the research was conducted in the absence of any commercial or financial relationships that could be construed as a potential conflict of interest.
